# Evolutionary Stabilization of Cooperative Toxin Production through a Bacterium-Plasmid-Phage Interplay

**DOI:** 10.1128/mBio.00912-20

**Published:** 2020-07-21

**Authors:** Stefanie Spriewald, Eva Stadler, Burkhard A. Hense, Philipp C. Münch, Alice C. McHardy, Anna S. Weiss, Nancy Obeng, Johannes Müller, Bärbel Stecher

**Affiliations:** a Max von Pettenkofer Institute of Hygiene and Medical Microbiology, Faculty of Medicine, LMU Munich, Munich, Germany; b German Center for Infection Research (DZIF), partner site LMU Munich, Munich, Germany; c Zentrum Mathematik, Technische Universität München, Munich, Germany; d Institute of Computational Biology, Helmholtz Zentrum München, Neuherberg, Germany; e Department for Computational Biology of Infection Research, Helmholtz Center for Infection Research, Brunswick, Germany; Hospital Ramon y Cajal; Max Planck Institute for Marine Microbiology

**Keywords:** bacteriophage, lysogen, virus, evolution, toxin, bacteriocin, regulation, heterogeneity, adaptive dynamics, evolutionary stable strategy, spiteful interaction, bistability, cheater, colicin, gastrointestinal infection, phenotypic noise

## Abstract

Colicins are toxins produced and released by *Enterobacteriaceae* to kill competitors in the gut. While group A colicins employ a division of labor strategy to liberate the toxin into the environment via colicin-specific lysis, group B colicin systems lack cognate lysis genes. In Salmonella enterica serovar Typhimurium (*S.* Tm), the group B colicin Ib (ColIb) is released by temperate phage-mediated bacteriolysis. Phage-mediated ColIb release promotes *S.* Tm fitness against competing Escherichia coli. It remained unclear how prophage-mediated lysis is realized in a clonal population of ColIb producers and if prophages contribute to evolutionary stability of toxin release in *S.* Tm. Here, we show that prophage-mediated lysis occurs in an *S.* Tm subpopulation only, thereby introducing phenotypic heterogeneity to the system. We established a mathematical model to study the dynamic interplay of *S.* Tm, ColIb, and a temperate phage in the presence of a competing species. Using this model, we studied long-term evolution of phage lysis rates in a fluctuating infection scenario. This revealed that phage lysis evolves as bet-hedging strategy that maximizes phage spread, regardless of whether colicin is present or not. We conclude that the ColIb system, lacking its own lysis gene, is making use of the evolutionary stable phage strategy to be released. Prophage lysis genes are highly prevalent in nontyphoidal *Salmonella* genomes. This suggests that the release of ColIb by temperate phages is widespread. In conclusion, our findings shed new light on the evolution and ecology of group B colicin systems.

## Abstract (precis)

Bacteria are excellent model organisms to study mechanisms of social evolution. The production of public goods, e.g., toxin release by cell lysis in clonal bacterial populations, is a frequently studied example of cooperative behavior. Here, we analyze evolutionary stabilization of toxin release by the enteric pathogen *Salmonella*. The release of colicin Ib (ColIb), which is used by *Salmonella* to gain an edge against competing microbiota following infection, is coupled to bacterial lysis mediated by temperate phages. Here, we show that phage-dependent lysis and subsequent release of colicin and phage particles occurs only in part of the ColIb-expressing *Salmonella* population. This phenotypic heterogeneity in lysis, which represents an essential step in the temperate phage life cycle, has evolved as a bet-hedging strategy under fluctuating environments such as the gastrointestinal tract. Our findings suggest that prophages can thereby evolutionarily stabilize costly toxin release in bacterial populations.

## INTRODUCTION

The gastrointestinal microbial ecosystem is shaped by competitive as well as cooperative interactions among and within bacterial populations. Cooperative mechanisms include mutualism with direct positive fitness consequences for both actors and recipients and altruism where the direct fitness consequence for the actor is negative but beneficial for the recipient (1). Colicin production and in particular lethal release are well-studied examples for altruistic behavior in bacterial populations (2). Colicins are protein toxins that mediate competitive exclusion of close relatives within the *Enterobacteriaceae* family (3, 4). Colicinogenic strains pay a high cost, as the toxin is released by bacteriolysis, thus ultimately leading to death of the producing bacterium. Colicinogenic populations solve this conflict via phenotypic heterogeneity: only a fraction of the population produce and release the colicin, while the remaining clonemates benefit from colicin as a public good. Evolution of cooperation is favored by mechanisms that ensure that benefits preferentially accrue to individuals that also carry genes for cooperation (1). Cooperation in bacterial populations by colicinogeny was shown to be favored in structured habitats. Here, compared to well-mixed systems, colicinogenic colonies generate locally increased colicin levels that effectively harm sensitive competitors, which provides a direct growth benefit for their clonemates (5, 6). Furthermore, horizontal transmission of altruistic genes to nonproducers can also sustain cooperation by increasing genetic similarity and thereby the direct and indirect benefit of public good transactions (7, 8). Indeed, the majority of colicins are carried on conjugative and mobilizable plasmids, suggesting that horizontal transmission plays a role in evolutionary stabilization of colicin production.

Colicins are divided in two groups (A and B) that differ in a number of characteristics, including operon structure, plasmid type, and mode of entry into the target cell. Most notably, group A colicin systems encode a specific lysis protein which is generally lacking in group B colicins. In group A colicin systems, colicin production is inevitably coupled to colicin release by cell lysis. Therefore, colicin expression is tightly regulated (9) and takes place only in a subpopulation that produces and releases colicin by cell lysis. Individual bacteria switch on toxin expression stochastically due to the regulation via the noisy SOS response (10, 11). Once a cell has turned on colicin expression, it produces the toxin for a short time and subsequently releases the toxin into the environment upon cell lysis (12). Especially at low cell numbers, stochastic switching can have important consequences on population dynamics and thereby determine competition outcome of colicin-producing and -sensitive strains (13).

**FIG 1:**
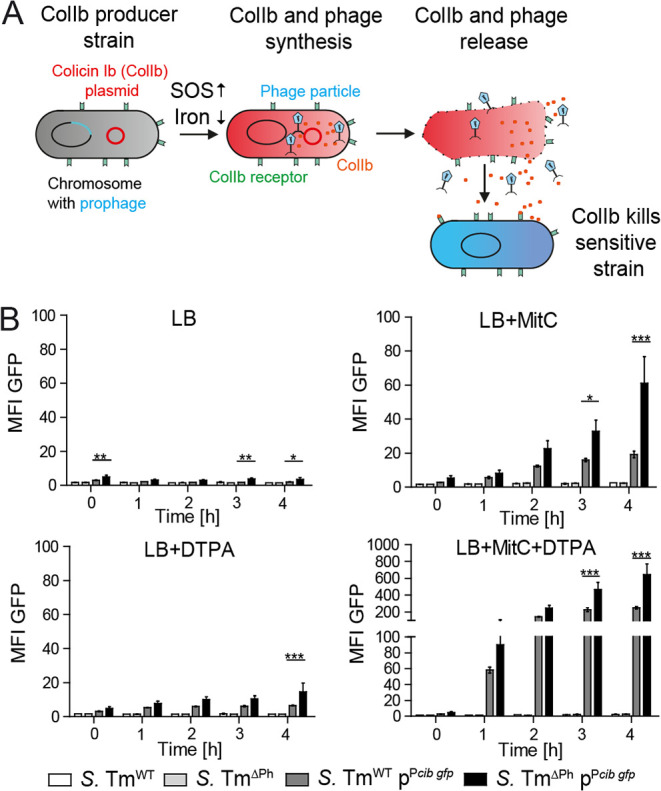
Individual ColIb+ Salmonella enterica serovar Typhimurium (*S.* Tm) lyse by temperate phage-mediated cell lysis in response to SOS stress. (A) The schematic diagram shows that ColIb release in *S.* Tm is mediated by λ-like prophages. (B) Mean fluorescence intensity (MFI) levels of *S.* Tm^WT^ p^P^*^cib gfp^* and *S.* Tm^Ph^ p^P^*^cib gfp^* and their respective negative controls *S.* Tm^WT^ and *S.* Tm^Ph^ were determined by flow cytometry. Bacteria were grown in LB media containing respective supplements (0.5 μg/ml MitC; 100 μM DTPA) for 4 h. The OD_600_ was monitored hourly, and samples were taken for flow cytometric analysis. Data from three independent experiments are shown. Statistical differences determined using two-way ANOVA with Bonferroni’s posttest (***, *P* < 0.001). Bars represent means and standard deviations (SD) (error bars).

Group B colicins lack a cognate lysis protein, and their mode of release was unclear for a long time (4). We previously reported a release mechanism for colicin Ib (ColIb), a prototype group B colicin, in a human-pathogenic Salmonella enterica serovar Typhimurium (*S.* Tm) strain (14). *S.* Tm SL1344 harbors a conjugative plasmid encoding ColIb (*cib*), which is expressed in response to iron limitation and the cell’s SOS response (15), conditions encountered by *S.* Tm during gut inflammation. Consequently, ColIb is expressed and provides the *S.* Tm population with a competitive advantage against competing Escherichia coli in the inflamed gut but not in the noninflamed gut (Fig. 1A). We demonstrated that the release of ColIb is mediated by λ-like prophages. Prophage-cured *S.* Tm strains and mutants in prophage lysis genes exhibit marked decline in environmental ColIb release and show reduced competitive advantage against ColIb-sensitive strains (14). Moreover, E. coli transformed with the ColIb plasmid gained the ability to release ColIb by lysogenization with a temperate phage (14). During lysogeny, temperate phages reside integrated within the *S.* Tm genome as prophages and are vertically transmitted by cell division. The lytic phase in λ-like prophages is induced by the bacterial SOS response (16), which eventually leads to host cell lysis and the concomitant release of ColIb and phage progeny (Fig. 1A). Thereby, prophage integration into the bacterial genome increases ColIb-dependent fitness of *S.* Tm (lysogenic conversion) in competition against closely related species, such as E. coli.

The recently discovered link between colicins and temperate phages added a new parameter to the ecology and evolution of colicin production (17). Several questions still remained unanswered. How is prophage-mediated lysis and ColIb production coordinated in *Salmonella* at the single-cell level? What is the frequency of ColIb and prophage lysis genes in human-pathogenic *Salmonella* strains? On the basis of the results of our previous experimental studies (14), we hypothesized that temperate phages introduce phenotypic heterogeneity in lysis expression and convey evolutionary stability of colicin release in their host bacteria. Here, we experimentally and theoretically investigate the dynamics of phage-mediated colicin release in *S.* Tm and address the co-occurrence of colicin and lysis genes families in the Salmonella enterica species.

## RESULTS

### Single-cell analysis of prophage-mediated *S.* Tm lysis under colicin-inducing conditions.

We previously showed that the gene encoding ColIb (*cib*) in *S*. Tm is unimodally expressed in the population upon induction (18). This observation was somewhat surprising, as in contrast, group A colicins are expressed bimodally, fitting a division of labor strategy (10, 11). Since ColIb is released by prophage-mediated lysis, we hypothesized that this mechanism may introduce stochasticity and consequently heterogeneous population behavior. To address this, we first quantified expression of ColIb (*cib*) and prophages at the single-cell level using a set of fluorescent gene reporters (see Tables S1 and S2 in the supplemental material). *S.* Tm SL1344 harbors four prophages: the lambdoid prophages Gifsy-1, Gifsy-2, and ST64B as well as the P2-like prophage SopEΦ (14). In the wild-type S. Tm (*S.* Tm^WT^), prophages are induced by the SOS response upon addition of the DNA-damaging drug mitomycin C. A *gfp* reporter plasmid (p^P^*^cib gfp^*), reporting ColIb production at the single-cell level (18) was transformed into wild-type *S.* Tm SL1344 (*S.* Tm^WT^) and prophage-cured (*S.* Tm^ΔPh^) cells (Tables S1 and S2). Bacteria were grown in liquid culture over the course of 4 h under ColIb/prophage-inducing (iron-chelatin diethylenetriaminepentaacetic acid [DTPA] and mitomycin C, see Materials and Methods) and noninducing conditions, and *gfp* expression was analyzed by fluorescence-activated cell sorting (FACS). Prophage-deficient *S.* Tm^ΔPh^ p^P^*^cib gfp^* exhibited substantially higher green fluorescent protein (GFP) mean fluorescence intensities (MFI) compared to *S.* Tm^WT^ under all investigated conditions, particularly in the presence of mitomycin C (Fig. 1B). A size-dependent effect regarding the differences in the MFI values between *S.* Tm^WT^ p^P^*^cib gfp^* and *S.* Tm^ΔPh^ p^P^*^cib gfp^* was ruled out by plotting the cell size (forward scatter [FSC]) against the MFI (see Fig. S1 in the supplemental material). This observation confirmed our previous data (14) indicating a scenario where ColIb (or GFP in this case) is not released in the absence of prophages and therefore accumulates inside the bacteria.

10.1128/mBio.00912-20.1 FIG S1 Differences in GFP signal intensity levels of *S.* Tm^WT pP^*^cib gfp^* compared to *S.* Tm^Δ^*^Ph^* p*^Pcib gfp^* are independent of cell size. Examples of dot-plots (forward scatter [FSC]/GFP) obtained from the experiment described in Fig. 1 demonstrate that *S.* Tm^Δ^*^Ph^* p*^Pcib gfp^* (black) exhibits higher, but size-independent (FSC) GFP signal intensity compared to *S.* Tm^WT pP^*^cib gfp^* (gray). Download FIG S1, PDF file, 1.3 MB. Copyright © 2020 Spriewald et al. 2020 Spriewald et al. This content is distributed under the terms of the Creative Commons Attribution 4.0 International license.

10.1128/mBio.00912-20.4 TABLE S1 Bacteria and plasmids used in this study. Download Table S1, PDF file, 0.1 MB. Copyright © 2020 Spriewald et al. 2020 Spriewald et al. This content is distributed under the terms of the Creative Commons Attribution 4.0 International license.

10.1128/mBio.00912-20.5 TABLE S2 Primers used in this study. Download Table S2, PDF file, 0.04 MB. Copyright © 2020 Spriewald et al. 2020 Spriewald et al. This content is distributed under the terms of the Creative Commons Attribution 4.0 International license.

**FIG 2:**
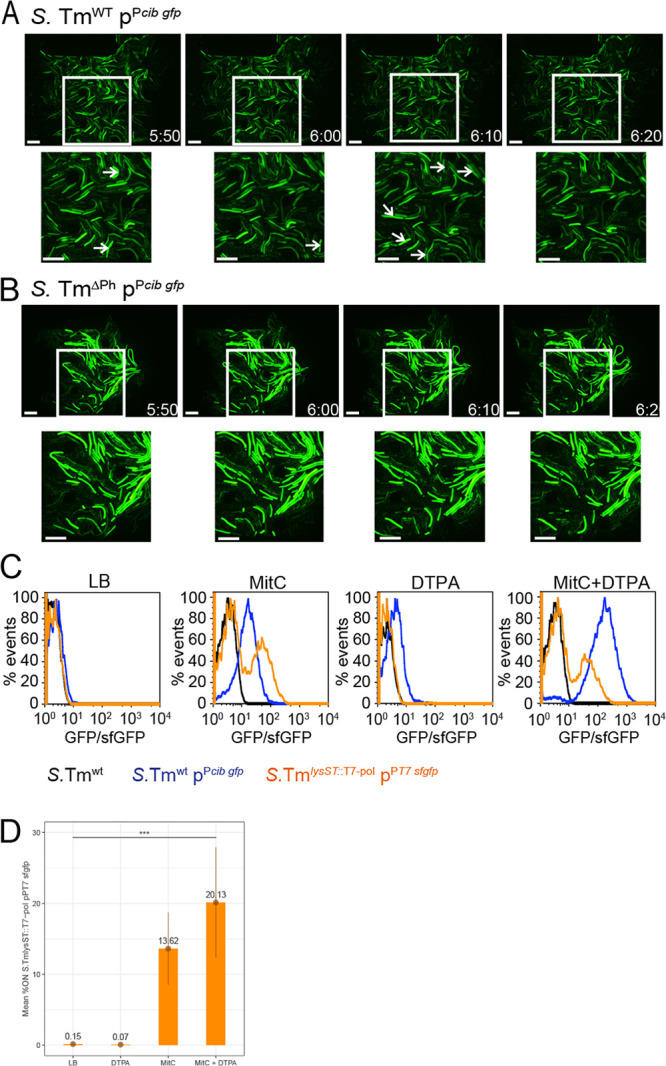
A fraction of the ColIb-producing *S*. Tm population simultaneously expresses ST64B prophage lysis genes. (A and B) Live-cell microscopy using the Onix microfluidic system (Millipore). Microcolonies of *S.* Tm^WT^ p^P^*^cib gfp^* (A) and prophage-deficient *S.* Tm^Ph^ p^P^*^cib gfp^* (B) were cultured in microfluidic plates for 3 h in LB only. Subsequently, medium was exchanged with LB supplemented with 100 μM DTPA for 20 min to induce *cib* expression. Next, LB supplemented with 100 μM DTPA and 0.5 μg/ml MitC was applied to induce the bacterial SOS response and full expression of *cib* and induce the prophage(s). After 20 min, cells were again exposed to LB supplemented with 100 μM DTPA for the remaining time of the experiment. Microscopic images obtained by live-cell microscopy (GFP-channel only) of *S.* Tm^WT^ p^P^*^cib gfp^* (A) and prophage-deficient *S.* Tm^Ph^ p^P^*^cib gfp^* (B) microcolonies. White arrows point to bacteria that are about to lyse. (C) *S.* Tm^WT^ p^P^*^cib gfp^* (blue) and *S.* Tm*^lysST^*^::T7 pol^ p^PT7^ *^sfgfp^* (orange) were grown in a 96-well plate in either LB with respective supplements (0.5 μg/ml MitC; 100 μM DTPA) for 4 h. Fluorescence signal intensities of GFP (*cib*) and sfGFP (ST64B prophage lysis genes) were determined by flow cytometric analysis, respectively. Background fluorescence signals of *S.* Tm^WT^ (black), grown under the same conditions, are shown as control. (D) Quantification of percent GFP (*cib*) “ON” cells from three replicate FACS experiments as described above for panel C.

Next, we investigated the *gfp* expression dynamics of individual *S.* Tm^WT^ p^P^*^cib gfp^* and prophage-cured *S.* Tm^ΔPh^ p^P^*^cib gfp^* cells under ColIb/prophage-inducing conditions using time-lapse microscopy in a CellASIC ONIX microfluidic platform. *S.* Tm microcolonies were grown in microfluidics chambers under ColIb-inducing conditions. Notably, lysis of individual *S.* Tm^WT^ cells was observed already within 2 h after mitomycin C exposure, while a part of the *cib*-expressing population did not lyse until the end of the measurement (Fig. 2A; see also Movie S1 in the supplemental material). In contrast, no lysis was observed in case of prophage-cured *S.* Tm^ΔPh^, even >3 h after mitomycin C exposure (Fig. 2B; Movie S2).

10.1128/mBio.00912-20.8 MOVIE S1 CellASIC live-cell microscopy of *S.* Tm^WT^p^Pcib gfp^. Using the CellASIC ONIX microfluidic system (Millipore), microcolonies of S. Tm^WT^p^P^*^cib gfp^* were initially grown in LB for 3 h. Subsequently, the medium was exchanged against LB supplemented with DTPA (100μM) to induce *cib* (*gfp*) expression only. Twenty minutes later, LB medium containing DTPA (100 μM) and MitC (0.5 μg/ml) was applied to induce full expression of *cib* (*gfp*) and in addition prophage-mediated cell lysis for 20 min. Afterwards, the medium was changed back to LB supplemented with DTPA (100 μM) only for the remaining time. (A) Merged bright-field and GFP channel. (B) GFP channel only. Download Movie S1, AVI file, 6.8 MB. Copyright © 2020 Spriewald et al. 2020 Spriewald et al. This content is distributed under the terms of the Creative Commons Attribution 4.0 International license.

10.1128/mBio.00912-20.9 MOVIE S2 CellASIC live-cell microscopy of *S.* Tm^ΔPh^p^P^*^cib gfp^*. Using the CellASIC ONIX microfluidic system (Millipore), microcolonies of *S.* Tm^ΔPh^p^P^*^cib gfp^* were initially grown in LB for 3 h. Subsequently, the medium was exchanged against LB supplemented with DTPA (100 μM) to induce *cib* (*gfp*) expression only. Twenty minutes later, LB medium containing DTPA (100μM) and MitC (0.5μg/ml) was applied for 20 min to induce full expression of *cib* (*gfp*) and to show the failure of cell lysis in the absence of prophages. Afterwards, the medium was changed back to LB supplemented with DTPA (100 μM) only for the remaining time. (A) Merged bright-field and GFP channel. (B) GFP channel only. Download Movie S2, AVI file, 6.5 MB. Copyright © 2020 Spriewald et al. 2020 Spriewald et al. This content is distributed under the terms of the Creative Commons Attribution 4.0 International license.

### Prophages introduce phenotypic heterogeneity in *S.* Tm lysis.

On the basis of the results of the time-lapse microscopy analysis, we reasoned that phenotypic heterogeneity in the ColIb system is introduced at the level of prophage-mediated cell lysis and therefore colicin release, rather than at the level of *cib* expression. *S.* Tm prophage ST64BΦ is from *S.* Tm ST64BΦ is a lambdoid phage often found in *S.* Tm virulent strains and of major importance in ColIb release (14, 19, 20). A double reporter strain (*S.* Tm*^lysST::T7 pol^* p^P^*^T7 rfp^* ^P^*^cib gfp^*) was constructed to simultaneously monitor ColIb production and induction of ST64BΦ lysis genes on the single-cell level (Tables S1 and S2). This strain relies on a signal amplification system based on the T7 polymerase gene *T7 pol*, integrated in the ST64B prophage lysis genes, and a plasmid-encoded red fluorescent reporter gene *tagRFP-T* under the control of the T7 promoter in addition to the ColIb *gfp* reporter. Therefore, red fluorescence indicates activation of ST64BΦ lysis genes and GFP indicates the induction of ColIb expression in the same bacterial cell. This reporter unequivocally shows that only a small fraction of ColIb-producing (GFP-positive [GFP^+^]) cells also expressed the lysis genes (tag RFP-T ^+^; Fig. S2 and Movies S3 and S4). In order to quantify the relative frequency of ST64B prophage lysis gene induction in *S.* Tm via FACS analysis, we generated another reporter strain, *S.* Tm*^lysST::T7 pol^* p^P^*^T7sfgfp^* (Tables S1 and S2). Again, signal amplification is based on the T7 polymerase gene *T7 pol*, integrated in the ST64B prophage lysis genes, and a T7 promoter-superfolder *gfp* (*sfgfp*) fusion on a plasmid. Therefore, ST64BΦ lysis gene induction is now monitored via *sfgfp* expression. Using this reporter strain and *S*. Tm^WT^ p^P^*^cib gfp^* (Fig. 1B and Fig. S1), it was confirmed that under ColIb/prophage-inducing conditions (DTPA, mitomycin C), ST64BΦ lysis genes are expressed only in a subset of the *S.* Tm population (20.13%), while *cib* is expressed unimodally (Fig. 2C and D) in about 80% of the population, as shown previously (18). In summary, using different reporter strains, it was shown that *S.* Tm expresses *cib* unimodally, while prophage lysis genes are expressed bimodally.

10.1128/mBio.00912-20.2 FIG S2 Examples of live-cell microscopy showing ST64B prophage lysis gene, and *cib*-expressing individual *S.* Tm*^lysST^*^::T7 pol^ bacteria. Initially, microcolonies of *S.* Tm*^lysST^*^::T7 pol^ p^PT7^ *^rfp^* ^P^*^cib gfp^* were cultured in microfluidic plates for 3 h in LB only. Subsequently, the medium was exchanged against LB supplemented with 100 mM DTPA for 20 min to induce *cib* expression of bacteria. Next, LB supplemented with 100 mM DTPA and 0.5 μg/ml mitomycin C was applied to induce the bacterial SOS response and thus full expression of *cib* and the expression of ST64B prophage lysis genes. After 20 min, the medium was changed back to LB supplemented with 100 mM DTPA only for the remaining time of the experiment. Images show that only a small fraction of the *S.* Tm*^lysST^*^::T7 pol^ p^PT7^ *^rfp^* ^P^*^cib gfp^* population expresses *cib* (*gfp*) and ST64B prophage lysis genes (*rfp*) simultaneously. Download FIG S2, PDF file, 1.9 MB. Copyright © 2020 Spriewald et al. 2020 Spriewald et al. This content is distributed under the terms of the Creative Commons Attribution 4.0 International license.

10.1128/mBio.00912-20.10 MOVIE S3 CellASIC live-cell microscopy of *S.* Tm^lysST::T7 pol^p^PT7^ *^rfpPcib gfp^*. Using the CellASIC ONIX microfluidic system (Millipore), microcolonies of *S.* Tm^lysST::T7 pol^p^PT7^ *^rfpPcib gfp^* were initially grown in LB for 3 h. Subsequently, the medium was exchanged against LB supplemented with DTPA (100 μM) to induce *cib* (*gfp*) expression only. Twenty minutes later, LB medium containing DTPA (100 μM) and MitC (0.5 μg/ml) was applied for 20 min to induce full expression of *cib* (*gfp*) and ST64B prophage lysis gene induction (*rfp*). Afterwards, the medium was changed back to LB supplemented with DTPA (100 μM) only for the remaining time. (A) Merged GFP and RFP channel. (B) GFP (*cib* expression) channel only; (C) RFP (ST64B prophage lysis gene expression) channel only. Download Movie S3, AVI file, 10.1 MB. Copyright © 2020 Spriewald et al. 2020 Spriewald et al. This content is distributed under the terms of the Creative Commons Attribution 4.0 International license.

### ColIb production does not affect bacterial growth rate.

Although the ColIb system does not mediate costly cell lysis itself, ColIb production may have a negative impact on *S.* Tm fitness by imposing a metabolic burden. Moving to study the effect of ColIb production on *S.* Tm growth, we extended our strain tool set. For this, we used *S.* Tm strain ATCC 14028 (*S.* Tm^WT 028^) as the basis because this strain originally does not carry a ColIb plasmid. From there, we generated a derivative carrying ST64BΦ only (*S.* Tm^WT 028^ *^ΔGif 1^*^,^*^2^*^,^*^3^*) and a prophage-deficient variant (S. Tm^WT 028^ *^ΔPh^*). All strains were transformed with pColIb (p2; Table 1) to explore the effect of ColIb on *S.* Tm fitness in different environments. We also cured the strains from P2 to address whether previous carriage of P2 had any effect on the fitness of the strains, e.g., by compensatory mutations. We grew cultures under control and various ColIb-inducing conditions and recorded optical density (optical density at 600 nm [OD_600_]) as a proxy for growth hourly. During 4 h of growth, the most prominent differences between WT and phage-deficient *S.* Tm strains appeared under SOS-inducing conditions (Fig. S3). Here, *S.* Tm^WT 028^ *^ΔGif 1^*^,^*^2^*^,^*^3^* carrying ST64BΦ only grew to slightly lower OD_600_ in the first 2 h of the experiment, followed by a sudden decline in OD_600_ after 2 h under SOS-inducing conditions. The prophage-deficient variant *S.* Tm^WT 028^ *^ΔPh^* showed positive growth rates over the entire 4 h. Notably, comparing the growth rates of strains with and without pColIb, we find no difference in any of the investigated conditions (Fig. S3). This suggests that carriage of pColIb and toxin expression does not measurably affect *S.* Tm growth rates.

**TABLE 1:** Expression patterns of *cib* compared to ST64B prophage lysis genes in individual bacteria, under noninducing and inducing conditions (Fig. S2)^*a*^

Time	MitC	DTPA	% ColIb^+^ (of total)	% ST64B lysis^+^ (of total)	% ColIb^+^ and ST64B lysis^+^ (of total)	% ST64B lysis^+^ (of ColIb^+^)
2 h	−	−	1.8	0.1	0	0
4 h	−	−	2.9	0.8	0	0
2 h	+	−	91.4	6.5	6	6.5
4 h	+	−	97.3	33.7	32.8	33.7
2 h	−	+	65.7	0.2	0.1	0.1
4 h	−	+	67.6	0.3	0.2	0.4
2 h	+	+	99.1	11.3	11	11.1
4 h	+	+	98.6	21.3	21.3	21.6

a The experiment has been replicated and yielded comparable results.

10.1128/mBio.00912-20.3 FIG S3 Net growth rates of *S.* Tm ^WT 028^ with and without prophages and pColIb. We compared growth rates of the WT strain (*S.* Tm^WT 028^), the strain containing only the phage ST64B (*S.*Tm^WT 028 ΔGif1,2,3^) and the prophage-free strain (*S.*Tm^WT 028 ΔPh^). Strains carried either no plasmid, plasmid pColIb (p2) or were again cured of the plasmid (p2^cured^). Growth rates were determined from changes in OD_600_ during growth experiments in 96-well plates. (A) As an example, growth of all strains over 4 h in LB (0.5 μg/ml MitC) is shown. (B to E) From growth curves, the change in biomass per hour was calculated as the slope of the linear regression of OD_600_ values before (hours 0 to 2) and after (hours 2 to 4) lysis induction. (B) LB medium, (C) iron-limiting conditions (100 μM DTPA), (D) SOS-inducing stress alone (0.5 μg/ml MitC) or in (E) combination with iron limitation (MitC/DTPA) are shown. Download FIG S3, PDF file, 0.2 MB. Copyright © 2020 Spriewald et al. 2020 Spriewald et al. This content is distributed under the terms of the Creative Commons Attribution 4.0 International license.

### Ecological model of the tripartite interaction of *S.* Tm, the colicin plasmid, and a temperate phage in a group B colicin system.

In the experiments described above, we demonstrated that prophage induction and subsequent ColIb release by cell lysis take place only in a subpopulation of ColIb-producing *S.* Tm. Even though ColIb increases the competitiveness of an *S.* Tm population (21), from an ecological perspective, colicin release by cell lysis is extremely costly. This presents a dilemma, as individuals then have an incentive to evade the cost of death or resulting lower growth rates due to colicin production, while the populations’ invasion of the gut relies on cooperative release of ColIb. Consequently, maintaining stability of intrastrain cooperation is a challenge for colicin-producing bacterial populations. As prophages contribute to ColIb release in our case, we wondered under which conditions phage-mediated colicin release is an evolutionary stable strategy in *S.* Tm.

**FIG 3:**
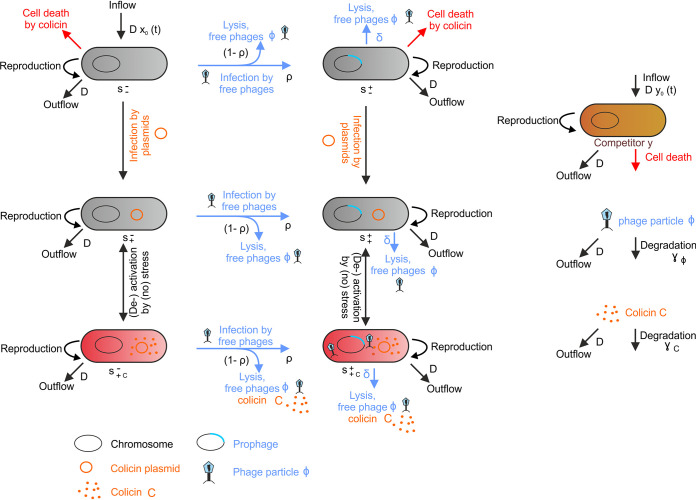
Transition graph for the ecological model of *S*. Tm, group B colicin plasmid and phage based on ordinary differential equations. The *S*. Tm population was structured into six compartments, according to the presence of prophage (right column), colicin plasmid (middle row) and induction of colicin expression and lysis (red cells, lower row). The *S*. Tm population is denoted with s, the superscript indicates the presence (+) or absence (−) of the prophage, and the subscript indicates the presence (+) or absence (−) of the plasmid. The additional *c* in the subscript indicates that the bacterium is producing colicin. $s_{-}^{-}$, *S*. Tm without colicin plasmid and without prophage; $s_{-}^{+}$, *S*. Tm without colicin plasmid and with prophage; $s_{+}^{-}$, *S*. Tm with colicin plasmid and without prophage, not producing colicin but immune to colicin; $s_{+}^{+}$, *S*. Tm with colicin plasmid and with prophage, not producing colicin but immune to colicin; $s_{+c}^{-}$, *S*. Tm with colicin plasmid and without prophage, producing and immune to colicin, no colicin release; $s_{+c}^{+}$, *S*. Tm with colicin plasmid and with prophage producing and immune to colicin, colicin release. Furthermore, free phages (Φ), free colicin (C), and a competitor population (*e*) are shown. See Text S1 in the supplemental material for further details of the model.

In order to study the dynamic interdependencies in this tripartite system of *Salmonella*, colicin, and a prophage during infection of the mammalian gut, we devised a model based on ordinary differential equations (ODEs) (Fig. 3; see also Text S1 in the supplemental material for details of the model). The model takes the following players into account: *S.* Tm (*s*), a plasmid encoding the colicin Ib (*cib*) and colicin Ib immunity (*imm*) genes, a temperate *S.* Tm phage, and a competing species (*e*), as, e.g., commensal E. coli (Fig. 3; Table S3). The mammalian gut is modeled as a chemostat with a periodic inflow of *S.* Tm (*s*_0_) to depict subsequent infections. The considered time scale of the interaction dynamics is ecological. Therefore, only *S.* Tm infection dynamics are addressed, while emerging *S.* Tm mutants are not considered. Even though *S.* Tm (*s*) may cause infections of different mammalian hosts in nature, pathogen host-to-host transition was not explicitly considered in the scale of this model.

10.1128/mBio.00912-20.6 TABLE S3 Parameter values used. Download Table S3, PDF file, 0.1 MB. Copyright © 2020 Spriewald et al. 2020 Spriewald et al. This content is distributed under the terms of the Creative Commons Attribution 4.0 International license.

10.1128/mBio.00912-20.11 TEXT S1 Additional information and equations for the modeling. Download Text S1, PDF file, 0.2 MB. Copyright © 2020 Spriewald et al. 2020 Spriewald et al. This content is distributed under the terms of the Creative Commons Attribution 4.0 International license.

ColIb can increase fitness of the *S.* Tm population in particular during inflammation-induced *Enterobacteriaceae* blooms, where *S*. Tm competes against close relatives (21). During blooms, the *S.* Tm population, as well as competing species, strongly expand due to increased availability of oxygen and altered nutritional environment (22). This effect was modeled as temporarily increased inflow D*s*_0_ (*t*) of all bacterial species. Furthermore, inflammation provides environmental cues (e.g., reactive oxygen species, iron limitation), which trigger ColIb production and prophage-mediated cell lysis and induce expression of the ColIb receptor (*cirA*) on competing E. coli (21, 23). Thus, we assumed that ColIb production pays off only when *S.* Tm grows in inflammation-induced blooms. To model inflammation-induced blooms, we further introduced periodic rounds of SOS signal as inducer of colicin expression and phage lysis (fluctuating environment).

Colicin plasmids and temperate phages act as “infectious” agents, which spread mainly by conjugation and the release of free phages Φ upon *S*. Tm host lysis, respectively (Fig. 3). *S.* Tm harboring the colicin plasmid ($s_{+}^{-}$) (Fig. 3, *s* row) are immune to the colicin, by expression of the cognate immunity protein. However, they do not produce colicin, unless there is an environmental trigger, as induction by SOS stress (Fig. 3, lower row) ($s_{+}^{-}$_c_). The temperate phage is integrated as prophage in the *S.* Tm genome ($s_{-}^{+}$). Prophage activation is induced by the bacterial SOS response as well. Therefore, in plasmid and prophage carrying *S*. Tm ($s_{+}^{+}$), SOS triggers lead to colicin production ($s_{+}^{+}$_c_), followed by lysis-mediated release of both colicin and free infectious phages (lysis rate δ). Upon infection of new hosts, the phage lyses new host cells or integrates into the bacterial genome with the probability ρ. Using parameter values determined from a combination of experimental data, literature research, and estimations (Table S3), the resulting ODEs describe the dynamics of the tripartite system of *Salmonella*, colicin, and a prophage and can now be used to study trait evolution.

### Adaptive dynamics analysis shows that phage-mediated lysis in the ColIb system is an evolutionary stable strategy.

Evolutionary stability of costly traits is often investigated by *in silico* competition experiments between players using prototypical, fixed opposing strategies. Thus, evolutionary stability of colicin release via phage-mediated lysis can be studied by investigating the competition of a wild-type strain with a mutant strain that does not lyse at all (17). However, in temperate phages, changes in the lysis rate occur by a stepwise process rather than by a drastic shift. For example, mutations in CI repressor operator binding sites can lead to subtle changes in lysis/lysogeny decisions (24). Evolutionary invasion analysis (adaptive dynamics) uses a set of mathematical modeling techniques to analyze the long-term evolution of a trait and thereby takes the stepwise adaptation of a strategy into account (25, 26). This type of model starts with an arbitrary wild-type (resident) strategy. From time to time, rare mutants (invaders, e.g., with an altered lysis rate) appear and attempt to invade the resident population and thereby might replace the wild type, leading to a change of the residents’ trait. Eventually, a stable state can be reached where no further mutant invasion is possible, which is referred to as the evolutionary stable strategy (ESS) of a trait. Instead of implementing a few prototypical strategies only, adaptive dynamics introduces a whole range of strategies and thereby reveals the direction in which evolutionary forces successively change a specific trait (convergent stability) (25, 26).

**FIG 4:**
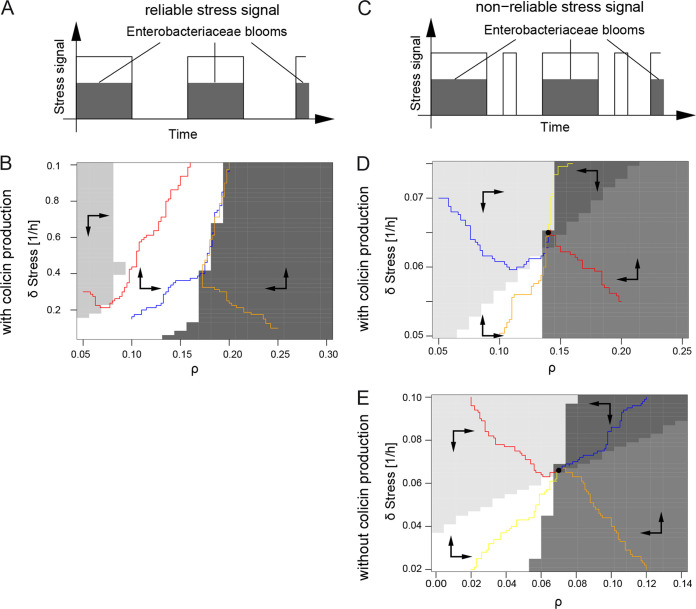
Evolutionary stability of the tripartite system of *S.* Tm, ColIb, and phage. Evolutionary stability of the tripartite system of *S.* Tm, ColIb, and phage was modeled when stress signals reliably (A and B) or nonreliably (C and D) indicate *Enterobacteriaceae* blooms. Inflammation-induced *Enterobacteriacea*e blooms are characterized by high abundance of *S.* Tm and potential competitors (e.g., E. coli). Environmental cues in blooms stimulate both induction of ColIb expression and prophages and represent a hot spot for colicin-dependent competition and phage transmission. (A) For the first case, it was assumed that the SOS signal (white bar) signals the occurrence of blooms (shaded bar) reliably, i.e., that ColIb and the phage are induced only in periods of *Enterobacteriaceae* blooms, when potential competitors are present at high abundance and colicin production really pays. (B) Pairwise invasibility plot (PIP) of joint evolution of lysis rate (δ_stress_) of lysogenic *S.* Tm and probability ρ to become lysogenic upon new infection by phages. The arrows within the gray areas indicate how the parameters are changed by evolution, the colored paths represent example paths of mutant populations. The PIP indicates that in case of a reliable signal, evolution drives δstress to arbitrarily large values. (C) In the second case, it was assumed that stress signals *Enterobacteriaceae* blooms nonreliably, i.e., that ColIb and the phage are also induced in periods in between blooms in the absence of competitors. (D and E) Resulting PIPs of joint evolution of lysis rate (δ_stress_) and probability to become lysogenic upon new infection of phages (ρ). The arrows within the gray areas indicate how the parameters are changed by evolution, the colored paths represent example paths of mutant populations, and the solid dot indicates the evolutionarily stable strategy (ESS). Specifically, panel D shows the full tripartite model, and panel E shows a model for only *S.* Tm and phage without colicin production and no competition. See Text S1 for parameters and details.

Using adaptive dynamics, we analyzed evolutionary stability of lysis from the phages’ perspective by invading mutant phages that have an altered lysis rate compared to the resident phage. Two different scenarios of phage-mediated lysis were considered. (i) Under stress conditions, prophage-mediated lysis of the lysogenic *S*. Tm population is induced by the SOS response at a given rate. (ii) Upon a new infection by phages, the majority of the *S*. Tm population lyses, and a certain fraction becomes lysogenic. Therefore, the dynamics of phage-mediated lysis are determined by two parameters, the lysis rate δ of lysogenic *S.* Tm and the probability ρ to become lysogenic. To investigate evolution of phage-mediated lysis in our ecological model, mutant phages with altered lysis rates δ_m_ and ρ_m_ were introduced at very small numbers, appearing as free infectious phages or as prophages in *S*. Tm. Using the resulting altered set of model equations, the Lyapunov exponent (rate of separation) was determined numerically for every adaptation step. If the Lyapunov exponent was positive, the mutant is able to spread; if it was negative, the mutant dies out. The resulting dynamics of the trait (given by the parameters δ and ρ) are presented as pairwise invasibility plots (PIP) (Fig. 4).

Using this approach, we investigated to what extent the reliability of an SOS signal as an indicator of *Enterobacteriaceae* blooms (representing an environmental condition, where colicin release will help to win against competitors) would influence the evolutionary fate of lysis. First, it was assumed that the SOS signal reliably indicates *Enterobacteriaceae* blooms, induced by inflammation (Fig. 4A). The resulting PIP indicates that in this scenario, evolution drives the lysis rate δ_stress_ to arbitrarily large values (Fig. 4B). If the signal was reliable, a higher lysis rate permits a more rapid release of phages during a bloom. As a result, this would allow a mutant with a higher lysis rate to spread and outcompete the wild type.

In the mammalian gut, other stimuli (drugs, metabolites) besides an inflammatory response could trigger the bacterial SOS response. Therefore, the SOS signal may not reliably indicate an *Enterobacteriaceae* bloom. For this reason, we implemented a second scenario, with an unreliable SOS signal (Fig. 4C). Here, an ESS exists for δ_stress_ > 0 and ρ > 0 (Fig. 4D). The resulting finite lysis rate reflects that stress is correlated with but not reliably signals inflammation. In this case, it is of advantage when a subpopulation of phage-infected *S*. Tm remains lysogenic, buffering the population from extinction. Thus, we conclude that the resulting ESS is a phage-mediated bet-hedging strategy caused by the unreliability of the SOS signal.

### Colicin release by *Salmonella* lysis is driven by the selfish nature of temperate phages.

We hypothesized that *S.* Tm temperate phage-dependent lysis is not optimized to release the colicin but to maximize the spread of the phages. To investigate whether colicin release is required to convey evolutionary stability in the phage lysis system, we implemented an alternative scenario without colicin release and without competitor. Notably, a positive lysis rate (δ_stress_ > 0) was still found to reach an ESS (Fig. 4E). Thus, the release of colicin by *S.* Tm lysis is not required to convey evolutionary stability of the temperate phage. Obviously, for temperate phages, bacterial death and the concomitant release of phage particles presents a private good. Our results indicate that the selfish behavior of temperate phages is the driving force for the ESS of *Salmonella* host lysis. The lysis-deficient ColIb system employs the evolutionary stable phage system to become evolutionary stable itself. This raises several interesting questions pertaining to the evolutionary relationship between the two systems, which remain open at the current stage of investigation.

### Distribution of colicins and phage lysis gene families on the genus Salmonella.

To gain insight into evolution of ColIb release by temperate phages, we next investigated the distribution of phage lysis genes and group B colicins in *Salmonella* strains more globally, using a genome-based approach. Salmonella enterica is the type species of the genus *Salmonella*. It comprises six subspecies and more than 2,500 serovars (27). At the time this analysis was performed, 9,499 genome sequences of S. enterica strains were available in the NCBI reference sequence (RefSeq) database, which we analyzed for the presence of colicin and lysis gene families (Table S4). To this end, we generated profile hidden Markov models (HMMs) for group A and group B colicins and phage lysis gene families and screened S. enterica chromosomal and plasmid sequences for matches. The rationale behind screening for phage lysis genes instead of all prophage-related sequences was to primarily capture functional prophages with intact lysis function. To account for the high sequence similarity of different colicin gene families, we filtered putative colicin matches to the best hit per genome. In case of phage lysis gene families, we allowed multiple hits.

10.1128/mBio.00912-20.7 TABLE S4 Gene families for group A and B colicins and phage lysis genes. Download Table S4, PDF file, 0.05 MB. Copyright © 2020 Spriewald et al. 2020 Spriewald et al. This content is distributed under the terms of the Creative Commons Attribution 4.0 International license.

**FIG 5:**
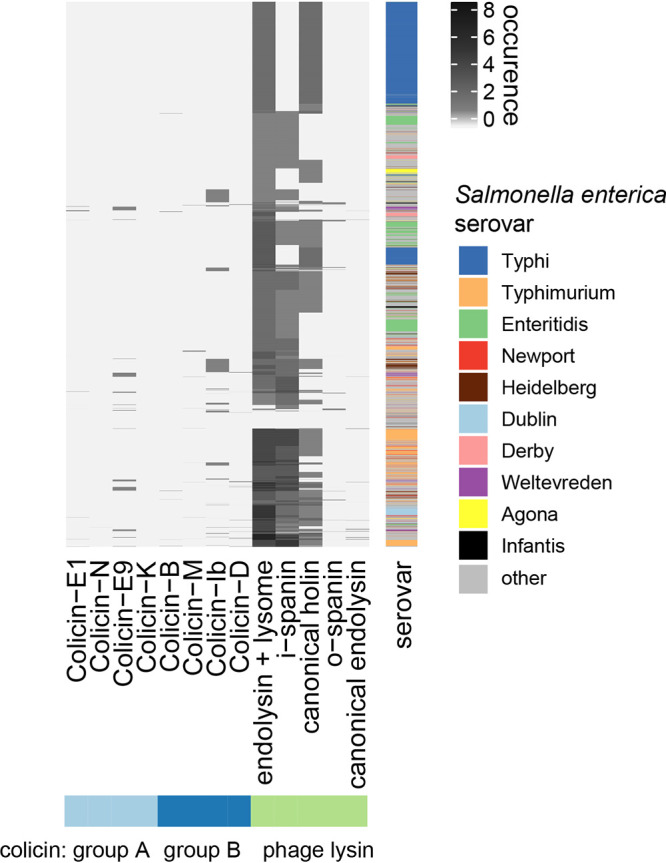
Distribution of colicin and lysis gene families in 9,499 S. enterica subsp. *enterica* genomes. The majority of S. enterica genomes contain phage lysis gene families, while colicin is present only in a subset of S. enterica genomes, quantified based on profile HMM matches. Mapping of colicin profile HMMs to S. enterica genomes are filtered based on the best hit, due to the high similarity of some colicin gene families. The 10 most prevalent serovars are annotated.

Prophages are widely distributed in the genus *Salmonella* (28). We also found that 9,196 of 9,499 (96.8%) S. enterica genomes have at least one lysis gene family in their chromosomal or plasmid sequence (in total 42,968 matches). Thus, only 303 genomes lack lysis genes at all. In total, 1,213 S. enterica genomes (12.8%) show matches to colicin HMM profiles (Fig. 5). Interestingly, genomes that are lysis positive (*n* = 9,196) seem to harbor colicin genes (group A and B) more often (12.9%, *n* = 1,183 with colicin match) than lysis-negative genomes (9.9.% of lysis-negative genomes, *n* = 30 with colicin match), but this difference is not significant (one-sided Fisher’s test, *P* = 0.072). Based on profile HMM matches, group B colicins are more abundant in S. enterica genomes (70.1%, *n* = 850) compared to group A colicins (29.9%, *n* = 363). We asked whether lysis-positive genomes (*n* = 9,196) harbor group B colicins more often than lysis-negative genomes (*n* = 303). In the subset of lysis-positive genomes, 9.0% (*n* = 830) harbor a colicin from group B, while this is only the case for 6.6% (*n* = 20) of lysis-negative genomes. Group A colicins are present in 3.8% (*n* = 353) of lysis-positive genomes and 3.3% (*n* = 10) of lysis-negative genomes. We conclude that B colicins are not more prevalent in Salmonella enterica genomes that do contain lysis genes (one-sided Fisher’s test, *P* = 0.084).

**FIG 6:**
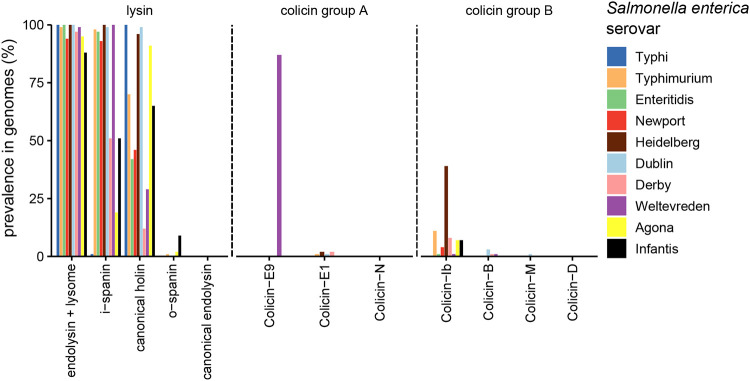
Prevalence of colicin and lysis gene families in S. enterica subsp. *enterica* serovars. Mapping of colicin profile HMMs to S. enterica genomes are filtered based on the best hit, due to the high similarity of some colicin gene families. Lysis-associated gene families are not filtered by the best hit and are stratified by the serovar. Prevalence of group A and group B colicins and phage lysins are shown for the 10 most common serovars, namely, serovars Typhi (*n* = 2142), Typhimurium (*n* = 1088), Enteritidis (*n* = 807), Newport (*n* = 285), Heidelberg (*n* = 267), Derby (*n* = 172), Dublin (*n* = 171), Weltevreden (*n* = 167), Agona (*n* = 145), and Infantis (*n* = 144).

Further, we stratified the Salmonella enterica genomes by serovar to check for serovar-specific differences in colicin and lysis gene abundances (Fig. 6). The frequency of group B colicins was highest in serovar Heidelberg, Typhimurium, and Derby (in this order). Notably, ColIb was the most prevalent group B colicin. ColIb is highly similar to ColIa at the protein level and cannot be distinguished based on an HMM. Thus, colicins annotated as ColIb in the analysis also comprise ColIa genes. Group A colicins were highly enriched in serovar Weltevreden (colicin E9), Heidelberg, and Derby (E1), but with limited occurrence. Interestingly, no colicins of any type were found in serovar Typhi. This could be accounted for by the fact that *S.* Typhi causes systemic rather than gastrointestinal infection, where it does not have to compete against the microbiota. Besides, serovar Typhi also did not harbor phage lysins of the spanin type. This suggests that serovar Typhi has a different spectrum of temperate phages. Overall, these data point at serovar-specific distribution and ecology of group A and B colicins in S. enterica.

Finally, we focused our analysis on serovar Typhimurium, our experimental model system. Notably, only a small set of the 1,088 genomes harbor group A colicins (0.74%, *n* = 8), while colicin group B is found in 11.8% (*n* = 128) of genomes. In serovar Typhimurium, lysis-associated gene families seem to be omnipresent (1,087 of all screened serovar Typhimurium genomes). This hindered us from analyzing whether group B colicins are also more frequently associated with prophages than group A. However, it suggests that due to high prevalence of prophages in serovar Typhimurium genomes, this serovar may generally be able to release and benefit from group B colicins.

## DISCUSSION

Colicins are widely distributed competition factors in *Salmonella* and E. coli. As colicin release benefits the population but is inevitably linked to death of the producer, colicins are a prime model to investigate how costly phenotypes are evolutionarily stabilized in bacterial populations. Particular attention has been paid to group A colicin systems, where colicin production is linked to bacterial lysis (13, 29, 30). In this study, we focused on the *Salmonella* ColIb system, where colicin release is not directly linked to ColIb production but realized by temperate phage-mediated lysis (14). It was still unknown how induction of phage-mediated lysis and colicin expression is coordinated at the single-cell level. Moreover, it remained unclear which role temperate phages play in evolutionarily stabilizing colicin release. We addressed these questions by combining single-cell analysis, mathematical modeling, and comparative genomics approaches.

Using *gfp* reporters and time-lapse microscopy, we show that temperate phage-mediated lysis occurs only in a fraction of the ColIb-producing *Salmonella* population. Confirming previous results by our group (18), *cib* is unimodally expressed in the *Salmonella* population. Thus, phenotypic heterogeneity in the ColIb system is introduced at the level of temperate phage-mediated lysis. In group A colicin systems, colicin and lysis genes are commonly cotranscribed. Thereby, colicin production is linked to bacterial lysis, and only a small fraction of the population actively express the toxin (4, 13). Additional regulatory mechanisms are in place to ensure that lysis only occurs with a time delay, once sufficient colicin has been synthesized within the bacteria (31, 32). Interestingly, colicin producers can stochastically switch between colicin silencing and expressing states, so colicin expression is not generally linked to lysis (29). In the ColIb system, a time delay between colicin expression and lysis is mediated by the complex regulatory cascade of the temperate phage lytic cycle. *S.* Tm strain SL1344 harbors four prophages with ΦST64B contributing most to ColIb release (14). ST64B lysis genes are expressed in ∼20% of the *cib*-expressing *S.* Tm population. The actual lysis rate of *S.* Tm under SOS stress is possibly higher due to the presence of the other prophages. Since induction of multiple prophages in polylysogenic strains is additionally controlled by complex cross-regulation (33), it is in fact hard to determine the contribution of the other *S.* Tm prophages to the overall lysis rate.

For temperate phages, a switch from the lysogenic stage to the lytic stage of the prophage occurs only in a subpopulation of lysogens (34–36). For *S.* Tm ΦST64B, the fractions of bacteria that induce lysis were 13% under SOS-inducing conditions and 20% when iron was additionally depleted (Fig. 2D). In the absence of stress, the spontaneous lysis frequency was low (0.15%). Interestingly, in E. coli, the Shiga toxin (Stx) phage, another lambdoid phage, even higher lysis frequencies of up to 60% of the E. coli population were reported, which varied by growth state of the host bacterium (37). This suggests that lysis rates of λ-like prophages exhibit a high degree in variability, which also strongly depends on the environmental conditions.

Using an evolutionary invasion analysis, *S*. Tm phages are expected to evolve a bet-hedging strategy. A subpopulation of *S*. Tm remains lysogenic and buffers the phage from extinction. Fluctuating environmental conditions are well-known to trigger bet-hedging. Typical examples are persister cells (38), sporulation (39), or seeds (40). The lytic-lysogenic pathways of temperate phages fit well into this scenario (23, 41). From an evolutionary perspective, temperate phages have an advantage over lytic phages in environments characterized by major fluctuations of their host bacteria (42). Under these conditions, the temperate phage can persist when host bacterial density is below the threshold required to sustain the propagation of the lytic population. According to the study of Maslov and Sneppen, successful phage strategies become more temperate in environments that are faced with frequent and severe downshifts of the host bacterial population (41). In the mammalian gut, *S.* Tm prophages are induced during periods of gut inflammation, when the chances for the phage are high to encounter new host bacteria (23). The same is actually true for ColIb, the expression of which is also triggered by signals in the inflamed gut (21). Apart from some exceptions (43), lysogenic phages cannot perceive their environment; we argue that regulation of phage lysis primarily responds to environmental cues directly or indirectly associated with the presence of closely related competing bacteria (e.g., *Enterobacteriaceae* blooms).

Maintaining evolutionary stability of cooperation by the release of public goods is a challenge for bacterial populations with phenotypic heterogeneity being an evolvable trait that can be shaped by natural selection. Theoretical and experimental analyses show that different mechanisms can maintain cooperative behaviors in microbial populations. These mechanisms include spatial proximity and local competition (44), pleiotropy (45), negative frequency-dependent selection (46), and collective behavior (47). We find that the ColIb system associates with an evolutionary stable trait (phage lysis). The coupling of two traits, where one trait directly or indirectly supports the other, is well-known in evolutionary theory. Neutral genes or genes encoding a cooperative phenotype in the vicinity of advantageous genes can spread in a population (48, 49).

Besides group B colicins, a number of other substances, including DNA and host-directed toxins are known to be released by temperate phage-mediated lysis (35, 50–52). On the one hand, the lytic/lysogenic phage system mediates lysis; on the other hand, the phenotype (colicin, Shiga toxin, DNA release) confers fitness benefits to the surviving population. It is conceivable that the substrate to be released can be readily exchanged. Thus, coupling traits to lysogenic phages might be a general mechanism to stabilize costly traits in bacterial populations.

Another well-known strategy that evolutionarily stabilizes colicin production is physical association of the cooperators and limited diffusion of the public good in a spatially structured environment (6, 53). This ensures that cooperators preferentially benefit from the released colicin. Group A colicins tend to establish a stable state in spatially structured environments (30, 54, 55). For the sake of simplicity, we assumed in our analysis that the gut is a rather well-mixed environment, despite microbial populations in different regions of the gut being highly structured (56). Notably, even in the absence of spatial structure, our model predicted that temperate phage-mediated lysis and ColIb release reaches an ESS. On the other hand, spatial structure may actually also hinder the spread of phage particles and infection of new hosts and limit the efficacy of toxins to target competitors. Therefore, it is not surprising that the *S.* Tm-ColIb-phage system forms an ESS in unstructured environments.

Plasmids conferring altruistic phenotypes come at higher costs and may be replaced by noncooperating mutant plasmids (7). Therefore, mechanisms conferring stability of the colicin plasmid can also contribute to the evolutionary stability of colicin release. First, colicin loci can function like toxin-antitoxin systems and can select for stable maintenance of colicin plasmids (57). Moreover, horizontal plasmid transfer to nonproducers can actually stabilize costly colicin production and release in the population (8, 58). Bacteria losing the plasmid can be reinfected and converted to producers. These strategies were primarily discussed for group A colicin plasmids that encode the lysis function itself. Besides this costly lysis, colicin expression itself may also be a burden for the bacteria. Our data suggest that ColIb plasmid does not affect *S.* Tm fitness, at least within short periods of time (see Fig. S3 in the supplemental material). In addition, it was previously shown that the ColIb plasmid is horizontally transferred to *S.* Tm in the gut environment with high efficiency (59). Therefore, it is conceivable that low costs and efficient spread of the ColIb plasmid explains the high prevalence of ColIb in the *S.* Tm population.

Release of ColIb is strongly enhanced by functional phage lysis genes in the colicin producer. Thus, horizontal transfer of a ColIb plasmid into a prophage-deficient strain would be an evolutionary dead-end, from the colicin’s perspective. Using comparative genome analysis, we find that prophage lysis functions are highly prevalent in nontyphoidal *Salmonella* serovars (NTS), in particular in serovar Typhimurium (Fig. 6). A recent study analyzed the prevalence and diversity of prophages in whole-genome sequences of 1,760 S. enterica strains using PHASTER. Similarly, prophage sequences were found most prevalent in serovar Typhimurium (148 strains; median ± interquartile range [IQR] of 9 ± 2 prophages per isolate) (28). This high prophage prevalence strongly suggests that *S.* Tm strains can release colicins, once they receive a corresponding plasmid.

The fact that the lysis is triggered by the phage and not the bacteria itself opens up many interesting questions. From the bacterium’s perspective, colicin release is clearly altruistic, while from the phagés perspective, lysis is an essential step of the replicative cycle and therefore a selfish act. Therefore, the question arises of whether bacterial altruism is not voluntary but enforced by the phage. There are examples of enforced altruism in insect societies (60). Alternatively, the evolutionary interests of the prophage and the bacterium are aligned—so it could be beneficial for both to induce lysis at the same time. After all, it remains an open question how ColIb dependency on phage lysis function has evolved. Clearly, several scenarios are conceivable. First, ancestral group B colicin plasmids may have carried lysis genes, but they were lost as lysis became a burden under changing environmental conditions. Subsequently, group B colicins relied on phage lysis functions for release. Alternatively, colicin lysis genes were rationalized because prophages became more prevalent and colicin was efficiently released in the course of phage lysis. In a third scenario, both, ancestral group A and group B colicins were released during phage lysis, and group A colicins evolved toward carrying their own lysis genes.

We conjecture that this difference between group A and group B colicins may be explained by ecological and environmental factors. Possible differences might be the localization of colicin-producing bacteria, e.g., in mucosal biofilms versus the gut lumen during *Enterobacteriaceae* blooms. Interestingly, we find that group B colicins were far more prevalent than group A colicins in NTS. Since NTS are well adapted to growth in inflammation-induced blooms (61) that select for a diverse range of *Enterobacteriaceae*, this may already indicate that group B colicins are more relevant in this environment (Fig. 5). Interestingly, NTS more frequently harbor colicins of any type compared to serovar Typhi. This serovar causes a systemic disease rather than colonizing the gut and is not well adapted to blooms (61).

In summary, our study reveals how ColIb expression and phage lysis are coordinated at the single-cell level and sheds new light on the evolutionary relationships between group B colicins and temperate phages in Salmonella enterica. It will be interesting to investigate whether similar interdependencies between temperate phages and chemical warfare functions exist in other bacterial species. Addressing this issue will contribute new insight into the diverse role of phages in mediating interspecies interactions in gut microbial communities.

## MATERIALS AND METHODS

### Generation of mutant strains and plasmids.

Bacterial strains and plasmids used in this study are listed in Table S1 in the supplemental material. The lambda red recombinase system described by Datsenko and Wanner (62) was utilized to generate *S.* Tm*^lysST::T7 pol^* (SJB34). For this purpose, plasmid pJLG2 was used as the template for the amplification of a *T7 gene1* (T7 polymerase gene). To generate *S.* Tm*^lysST::T7 pol^* (SJB34), oligonucleotides T7_ST64B_fwd/ST64B-sfgfp-rev (Table S2) were used to amplify the *T7 gene1* (T7 polymerase gene), including a FRT site-flanked kanamycin cassette, from the template plasmid pJLG2. Correct exchange of the ST64B prophage lysis genes (SL1344_1955 to SL1344_1957) against the reporter gene *T7 gene1* in SJB33 was verified by PCR and sequencing using the oligonucleotides Check_ST64_for/T7seq left 2/T7 seq right 2/Check_ST64_rev (Table S2). The allele *SL1344_1955-SL1344_1957*::*T7 gene1-aphT* of SJB33 was P22 transduced into a clean *S.* Tm^WT^ strain, resulting in strain SJB34 (*S.* Tm*^lysST::T7 pol^*). The functionality of this reporter was controlled by the transformation of pJLG1 (carrying a T7 promoter *sfgfp* fusion) into *S.* Tm*^lysST::T7 pol^*, following flow cytometry analysis.

To construct pM955, plasmid pM946 (63) was hydrolyzed with EcoRI and religated to remove *gfpmut3b*. Generation of p^PT7^ *^sfgfp^* (pJLG1) was achieved by amplification of the kanamycin resistance cassette-flanked *sfgfp* gene (encoded on pWRG7) using PCR and the oligonucleotides pJLG1 SFGFP SD BamHI Fw/pJLG1 SFGFP EcoRI Rev (Table S2). Insertion of the amplicon into pM955 was done by restriction hydrolysis with BamHI/EcoRI. Correct insertion was controlled by sequencing using the oligonucleotides pJLG1 proof fwd/pJLG1 proof rev. Initially, to generate template plasmid pJLG2 (carries the kanamycin resistance cassette-flanked *T7 gene1*), genomic DNA (gDNA) was extracted from E. coli BL21(DE3). Next, the T7 polymerase gene *T7 gene1* was amplified by PCR using the oligonucleotides T7 pol FW NotI/T7 pol Rev XhoI (Table S2) and inserted by restriction hydrolysis with NotI/XhoI into p2795 (64). Plasmid was confirmed by sequencing using the oligonucleotides pJLG2 seq Fw/T7 seq left 2/T7 seq right 2/pJLG2 seq Rev (Table S2).

In order to generate T7 promoter *tag rfp T* fusion reporter plasmid (pSJB26), a PCR was conducted with oligonucleotides TagRFP-T_Fwd2/TagRFP-T_Rev (Table S2) to amplify *tag rfp T* from the template plasmid pWRG435 (65). This amplicon was inserted in pM955 by restriction hydrolysis with BamHI/EcoRI. Correct insertion was controlled by sequencing using the oligonucleotides pJLG1 proof fwd/pJLG1 proof rev (Table S2). To generate the double reporter plasmid p^P^*^T7 rfp^* ^P^*^cib gfp^* pSJB28, the P*cib gfp* fusion, including the transcription terminator sequences *rrnbT1 rrnbT2* were removed from pM1437 and inserted in pSJB26 using the enzymes XhoI/SalI.

To transform strains with the colicinogenic plasmid pColIb-P9 (p2), genomic DNA was isolated from *S.* Tm M1407, a strain carrying a nonconjugative version of pColIb (pColIb*oriT-NikA*::cat [14]) and used for transformation in electrocompetent *S.* Tm strains. p2 was cured using a method described previously (59). Briefly, pM1451, harboring the origin of replication of p2 and the selection marker *bla*, was transformed by electroporation. Bacteria that replaced p2 by pM1451 were serially selected on agar plates and liquid culture containing ampicillin (100 μg/ml). A culturing step in LB medium without selective antibiotics then ensured the loss of pM1451. Resulting strains were all confirmed by PCR to be cured of p2.

### Bacterial growth conditions.

In general, bacterial strains were at first cultured in test tubes (Cultube sterile culture tubes, tubes with cap, polystyrene; 17 mm × 95 mm height [H]), containing 3 ml LB medium (supplemented with respective antibiotics) for 12 h at 37°C in a rotor wheel. This overnight (o.n.) culture was normalized to an optical density at 600 nm (OD_600_) of 0.5 and used to inoculate subculture I (ratio 1:20) in test tubes containing 3 ml LB (supplemented with respective antibiotics). Following an incubation time of 2 h at 37°C in a rotor wheel, subculture I was also normalized to an OD_600_ of 0.5. Normalized subculture I was used to inoculate subculture II. This was done in a 96-well plate containing LB medium only or LB supplemented with either mitomycin C (MitC) (final concentration, 0.5 μg/ml) or diethylenetriaminepentaacetic acid (DTPA) (final concentration, 100 μM) or both in a ratio 1:1. Incubation of subcultures II was carried out for 4 h at 37°C and 180 rpm.

### Growth for live-cell microscopy.

Initially, bacteria were streaked on LB agar plates containing appropriate antibiotics and incubated o.n. at 37°C. A single colony was used to inoculate 3 ml LB in test tubes (Cultube sterile culture tubes, tubes with cap, polystyrene; 17 mm × 95 mm H) and incubated for 12 h at 37°C in a rotor wheel. This o.n. culture was used to inoculate 2 ml LB medium (ratio 1:20). The subculture was incubated for 2 h in a rotor wheel at 37°C. Next, a bacterial sample of this subculture was taken and diluted in 1× phosphate-buffered saline (PBS) to achieve the concentration of 10^6^ bacterial cells/ml.

### Flow cytometry.

Bacteria were grown in 96-well plates as described before and diluted in 1× PBS to a concentration of 10^6^ CFU. Fluorescence-activated cell sorting (FACS) data were recorded by FACS CantoII running the FACSDiva software (Aria Becton Dickinson). The FlowJo software 8.8.4 (Tree Star, Inc.) was used for data analysis.

### Live-cell microscopy using CellASIC ONIX microfluidic platform.

The microfluidic plate for bacteria (B04; Millipore) was primed with LB medium. This was achieved by exchanging the buffer of the medium/buffer reservoir wells 1 to 5 against LB medium. The cell outlet well 6 and the waste well 7 were emptied. A drop of 50 μl of 1× PBS was placed in the bottom of well 7. The manifold was attached to the plate and by the CellASIC ONIX microfluidic perfusion system (model EV262); the plate was vacuum sealed and primed with LB medium of wells 1 to 5 simultaneously (5 lb/in^2^, 5 min). Next, the vacuum was removed, and wells 1 to 5 were filled with the test medium (300 μl/well): LB only, LB supplemented with 100 μM DTPA or LB supplemented with 0.5 μg/ml MitC and 100 μM DTPA. Finally, 100 μl of the cell suspension (10^6^ cells/ml) was loaded in the cell inlet well 8. The manifold was attached to the plate, and the plate was vacuum sealed and placed onto a spinning disk confocal microscope (TE300 eclipse; Nikon Instruments, Düsseldorf, Germany) with Perkin Elmer UltraVIEW spinning disk system and Hamatsu Orca ER charge-coupled-device (CCD) or Orca Flash 4.0 complementary metal oxide semiconductor (CMOS) camera. The microscope was surrounded by a climate chamber with a temperature of 37°C. Bacterial cells were loaded (4 lb/in^2^ for 6 s) into the system followed by an additional priming step with LB medium only (5 min, 5 lb/in^2^) to wash out untrapped bacteria. The CellASIC ONIX FG software was used to set up a protocol for the medium flow (Fig. 1B). In general, a pressure of 2 lb/in^2^ for 1 min was used to exchange the previous medium against the new medium; otherwise, a pressure of 1 lb/in^2^ was used for the medium flow. A Nikon 100×/1.40 Plan Apo oil objective was used to take images every 10 min.

### Statistical analysis.

Statistical analyses were performed with Graph Pad Prism version 5.01. Dependent on the analysis, the one-way analysis of variance (ANOVA) test followed by the Kruskal-Wallis test with Dunn’s multiple-comparison test or the two-way ANOVA with Bonferroni’s posttest was used. *P* values less than 0.05 were considered significant.

### Mathematical modeling.

We implemented the system of ordinary differential equations using the simulation package simbTUM (66). To allow for a fast simulation, the model was exported to the programming language C. The differential equations were solved by an implicit Euler procedure (with a Newton procedure and fixed step width). The solver was tested using a comparison with XPPaut (67) and R/package deSolve (68) for several equations. In order to estimate the Lyapunov exponent for a rare mutant, we solved the system with the resident only for four periods such that the system saddled down on a periodic solution. Then, the mutant was introduced at a small population size (ratio of mutant/resident population size, 10^−8^). After a second burn-in phase with the mutant (2.75 periods), the growth of the mutant was measured for 2 periods. For some arbitrary parameter sets, it was checked that mutants with a positive Lyapunov coefficient took over the population as required by adaptive dynamics. The data were analyzed with R (69).

### Genome-based analysis of colicin and lysis gene families.

A total of 9,499 Salmonella enterica genomes were downloaded from the NCBI Reference Sequence Database (on 12 December 2018) using the search term “Salmonella enterica” and filter for “Prokaryotes” (not filtered for assembly level) and used for the quantification of colicin and lysis gene families. For this analysis, profile hidden Markov models (profile HMMs) (70) were generated for 13 gene families, where five belong to phage lysis genes and eight to group A and B colicin genes. Individual sequences of gene families were downloaded from UniRef50 (71) (colicin) and Pfam (71) (lysis). UniRef terms were searched via UniProt (https://www.uniprot.org/uniref/). Amino acid sequences for colicin gene families were aligned using Clustal Omega (72) employing standard parameters. Profile HMMs of colicin gene families of the MSA were generated using the hmmbuild command of HMMER with standard parameters. The full HMM models for the Pfam collection (v. 32) were downloaded via EBI. We searched all genomes for homologs using hmmsearch from HMMER and filtered them based on alignment accuracy of >70%. Because of the high similarity of colicin profile HMMs, we filtered colicin matches further based on the best hit to each genome. Heatmaps were generated using complex heatmap library (73). We spot-checked the sensitivity of HMM matches using the .gff annotation from NCBI. Scripts and data to reproduce analysis are available at https://github.com/hzi-bifo/colicin-lysis.
