# Supplementary material for: Evolutionary Stabilization of Cooperative Toxin Production through a Bacterium-Plasmid-Phage Interplay
Source: mBio. 2020 Jul 21;11(4):e00912-20. doi: 10.1128/mBio.00912-20 (PMC7374059; doi:10.1128/mBio.00912-20)
Supplement: FIG S1 [file mBio.00912-20-sf001.pdf]

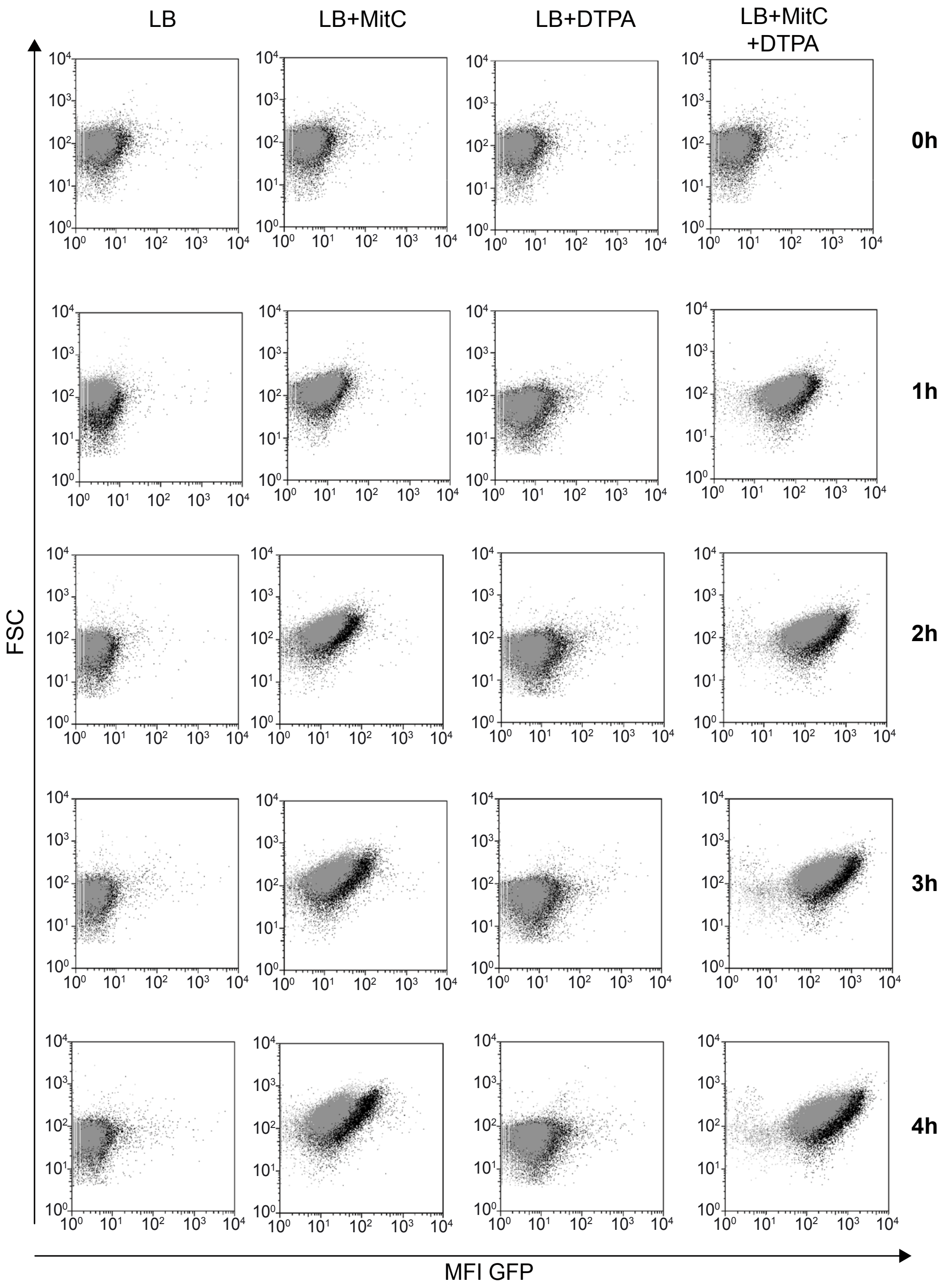

Fig. S1. Differences in GFP signal intensity levels of *S.* TM^{WT}p$^{P_{cib\ gfp}}$ compared to *S.* Tm$^{\Delta Ph}$p$^{P_{cib\ gfp}}$ are independent of cell size. Examples of dot-plots (FSC/GFP) obtained from the experiment described in Figure 1 demonstrate that *S.* Tm$^{\Delta Ph}$p$^{P_{cib\ gfp}}$ (black) exhibitsh igher, but size-independent (FSC) GFP signal intensity compared to *S.* TM^{WT}p$^{P_{cib\ gfp}}$ (grey).
