# Supplementary material for: Evolutionary Stabilization of Cooperative Toxin Production through a Bacterium-Plasmid-Phage Interplay
Source: mBio. 2020 Jul 21;11(4):e00912-20. doi: 10.1128/mBio.00912-20 (PMC7374059; doi:10.1128/mBio.00912-20)
Supplement: TABLE S1 [file mBio.00912-20-st001.pdf]

Table S1. Bacteria and Plasmids used in this study

***S.* Tm strains**	*Lab-internal strain ID*	*Relevant characteristics*	*Reference*
S. Tm^{WT}	SB300	*S.* Tm strain SL 1344, Strep^{R}	(?)
S. Tm^{WT}	MA6118	*S.* Tm wild type strain SL 1344, Strep^{R}	(?)
S. Tm$^{\Delta \mathrm{Ph}\ \Delta \mathrm{pRSF1010}}$	MA7891	MA6118, ΔGifsy-1 ΔGifsy-2 ST64B::*aphT*, SopEΦ::*cat*, ΔpRSF1010-SL 1344, Kan^{R}, Cm^{R}	(?)
S. Tm$^{\Delta \mathrm{Ph}}$	LPN27-1	MA7891, pRSR1010-SL 1344, Strep^{R}, Kan^{R}, Cm^{R}	(?)
S. Tm$^{lysST::T7\,pol}$	SJB34	SB300, ST64B (*SL 1344_1955 - SL 1344_1957*)::*T7 gene 1 aphT*, Strep^{R}, Kan^{R}	This study
S. Tm$^{\mathrm{WT}\ 028}$	ATCC 14028s	Wild type	(?)
S. Tm$^{\mathrm{WT}\ 028}$p2	NO1	ATCC 14028s, plasmid p2 Δ*oriT-NikA::cat*	This study
S. Tm$^{\mathrm{WT}\ 028}$p2^{cured}	NO8	ATCC 14028s, cured of plasmid p2 Δ*oriT-NikA::cat*	This study
S. Tm$^{\mathrm{WT}\ 028\ \Delta\ Gif1,2,3}$	MA6052	ATCC 14028s Δ *Gifsy-1,-2,-3*	(?)
S. Tm$^{\mathrm{WT}\ 028\ \Delta\ Gif1,2,3}$p2	NO2	MA6052, plasmid p2 Δ*oriT-NikA::cat*	This study
S. Tm$^{\mathrm{WT}\ 028\ \Delta\ Gif1,2,3}$p2^{cured}	NO9	MA6052, cured of plasmid p2 Δ*oriT-NikA::cat*	This study
S. Tm$^{\mathrm{WT}\ 028\ \Delta\ Ph}$	MA7549	ATCC 14028s, Δ*Gifsy-1,-2,-3*, Δ*ST64B*	(?)
S. Tm$^{\mathrm{WT}\ 028\ \Delta\ Ph}$p2	NO3	MA7549, plasmid p2, Δ*oriT-NikA::cat*	This study
S. Tm$^{\mathrm{WT}\ 028\ \Delta\ Ph}$p2^{cured}	NO10	MA7549, cured of plasmid p2, Δ*oriT-NikA::cat*	This study

***E. coli* strains**			
Ec$^{\mathrm{DH5}\alpha}$	DH5α	*F*$^{-}$, Φ*80lacZ*Δ*M15*, Δ(*lacZYA-argF*), *U169*, *recA1, endA1, hsdR17*(r_k^-, m_k^+), *phoA, supE44*, *thi-1, gyrA96, relA1*, λ^-	Invitrogen
Ec$^{\mathrm{MG1655}}$	MG1655	*E. coli* K12 wild type strain MG1655, F, λ, *ilvG, rfb-50, rph-1*	(?)
Ec$^{\mathrm{BL21(DE3)}}$	BL21 (DE3)	BL21 (DE3), *B F^{-} dcm ompT hsdS (rB^{-}mB^{-}) gal I (DE3)*	Stratagene

**Plasmids**	*Lab-internal plasmid number*	*Copy number*	*Relevant characteristics*	*Reference*
	pWKS30	low	pSC101 origin of replication, Amp^{R}	(?)
	pKD46	low	λ Red-expression under control of pBAD, temperature-sensitive, Amp^{R}	(?)
	pWRG7	medium	Promoterless *sfgfp-aphT* lambda red template vector, Kan^{R}	(?)
	pWRG435	medium	P$_{rprsM}$ *tag rfp T, oriT, mobA* Amp^{R}	(?)
	pCP20	low	*FLP*$^{+}$, λ cl857^{+}, λ *p*$_{\mathrm{R}}$ Rep^{ts}, Amp^{R}, Cm^{R}	(?)
	pM946	low	*ori pSC101*, pWKS30 cured of P$_{lac}$, P$_{\mathrm{T7}}$ *gfpmut3b*, Amp^{R}	(?)
	pM955	low	*ori pSC101*, pWKS30 cured of P$_{lac}$, Amp^{R}	This study
p$^{\mathrm{P}cib\,gfp}$	pM1437	medium	*ori pBR322, ori F1*, P$_{cib}$::*gfpmut2*, Amp^{R}	(?)
p$^{\mathrm{PT7}\,sfgfp}$	pJLG1	low	pM955, P$_{T7}$::*sfgfp*, Amp^{R}	This study
p$^{T7\,Pol}$	pJLG2	low	p2795, *T7 gene 1 aphT,* Amp^{R}, Kan^{R}	This study
	pSJB26	low	pM955, P$_{T7}$::*tag rfp T*, Amp^{R}	This study
p$^{\mathrm{PT7}\,rfp\,\mathrm{P}cib\,gfp}$	pSJB28	low	pSJB26, P$_{T7}$::*tag rfp T*, P$_{cib}$::*gfpmut2*, Amp^{R}	This study
p2		low	pColIB ΔoriT nikA::cat of *S.*Tm M1407	(?)
