# Supplementary material for: Evolutionary Stabilization of Cooperative Toxin Production through a Bacterium-Plasmid-Phage Interplay
Source: mBio. 2020 Jul 21;11(4):e00912-20. doi: 10.1128/mBio.00912-20 (PMC7374059; doi:10.1128/mBio.00912-20)
Supplement: TABLE S2 [file mBio.00912-20-st002.pdf]

Table S2. Primers used in this study

Designation	Sequence
Check_ST64_for	CTGTTTGGCGGCCTTTTC
Check_ST64_rev	GGA TAT AAA AAC GCC CCG
pJLG1 proof fwd	GGGGATGTGCTGCAAGG
pJLG1 proof rev	GAGCTGACTGGGTTGAAG
pJLG1 SFGFP SD BamHI Fw	GATCCTAAGGAGGGTAAGCATGCGCAAAGGCGAAG
pJLG1 SFGFP EcoRI Rev	GTAGAATTCTTATTATTTATACAGTTCATCCATGCCA
pJLG2 seq Fw	GCTCTGAAGTTCCTATACT
pJLG2 seq Rev	GCTATGACCATGATTACG
ST64B-sfgfp-rev	ACGAGGCATTTTCATGAAAGTCACTTGTCAAATTTCTA TGTGATGGAAACGTGTAGGCTGGAGCTGCTTC
T7 pol FW Notl	CATGCGGCCGCATGAACACGATTAACATCGCTAAG
T7 pol pol Rev Xhol	TCACTCGAGTTACGCGAACGCGAAGTC
T7 seq left 2	CTAAGGGTCTACTCGGTGGC
T7 seq right 2	TCACGCTCACAGATTCCCAA
T7_ST64B_fwd	TCCACTCACCCGATACCCGGGTAAACAGTCTCCCGGAC AGGGGGAGGTCATGAACACGATTAACATCGCTAAGAA
TagRFP-T_Fwd2	TCTGGATCCTAGATTTAAGAAGGAGATATACATATG
TagRFP-T_Rev	TCTGAATTCTTATTTATACAGTTCATCCATGCC
