# Supplementary material for: Evolutionary Stabilization of Cooperative Toxin Production through a Bacterium-Plasmid-Phage Interplay
Source: mBio. 2020 Jul 21;11(4):e00912-20. doi: 10.1128/mBio.00912-20 (PMC7374059; doi:10.1128/mBio.00912-20)
Supplement: FIG S2 [file mBio.00912-20-sf002.pdf]

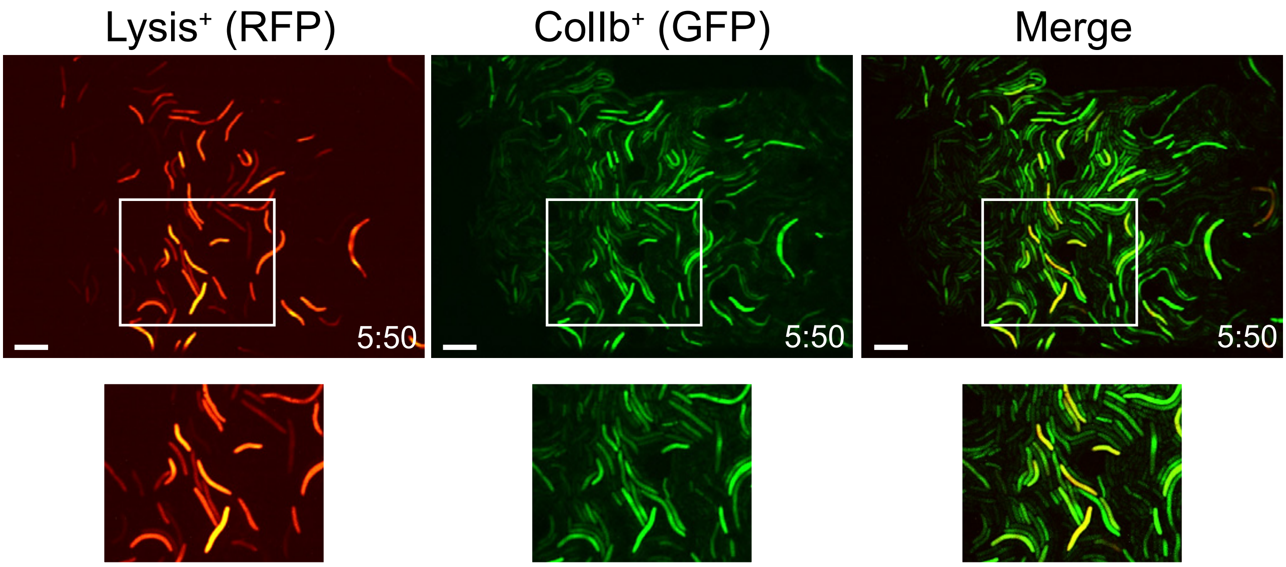

Fig. S2. Examples of live cell microscopy showing ST64B prophage lysis gene and *cib* expressing individual *S.* Tm$^{lysST::T7\ pol}$ bacteria. Initially, microcolonies of *S.* Tm$^{lysST::T7\ pol}$ p$^{\mathrm{PT7}\ rfp\ \mathrm{P}cib\ gfp}$ were cultured in microfluidic plates for 3 h in LB only. Subsequently, media was exchanged against LB supplemented with 100 μM DTPA for 20 min to induce *cib* expression of bacteria. Next LB supplemented with 100 μM DTPA and 0.5 μg/ml was applied to induce the bacterial SOS-response and thus full expression of *cib* and the expression of ST64B prophage lysis genes. After 20 min, the medium was changed back to LB supplemented with 100 μM DTPA only, for the remaining time of the experiment. Images show that only a small fraction of the *S.* Tm$^{lysST::T7\ pol}$ p$^{\mathrm{PT7}\ rfp\ \mathrm{P}cib\ gfp}$ population expresses *cib* (*gfp*) and ST64B prophage lysis genes (*rfp*), simultaneously.
