# Supplementary material for: Evolutionary Stabilization of Cooperative Toxin Production through a Bacterium-Plasmid-Phage Interplay
Source: mBio. 2020 Jul 21;11(4):e00912-20. doi: 10.1128/mBio.00912-20 (PMC7374059; doi:10.1128/mBio.00912-20)
Supplement: FIG S3 [file mBio.00912-20-sf003.pdf]

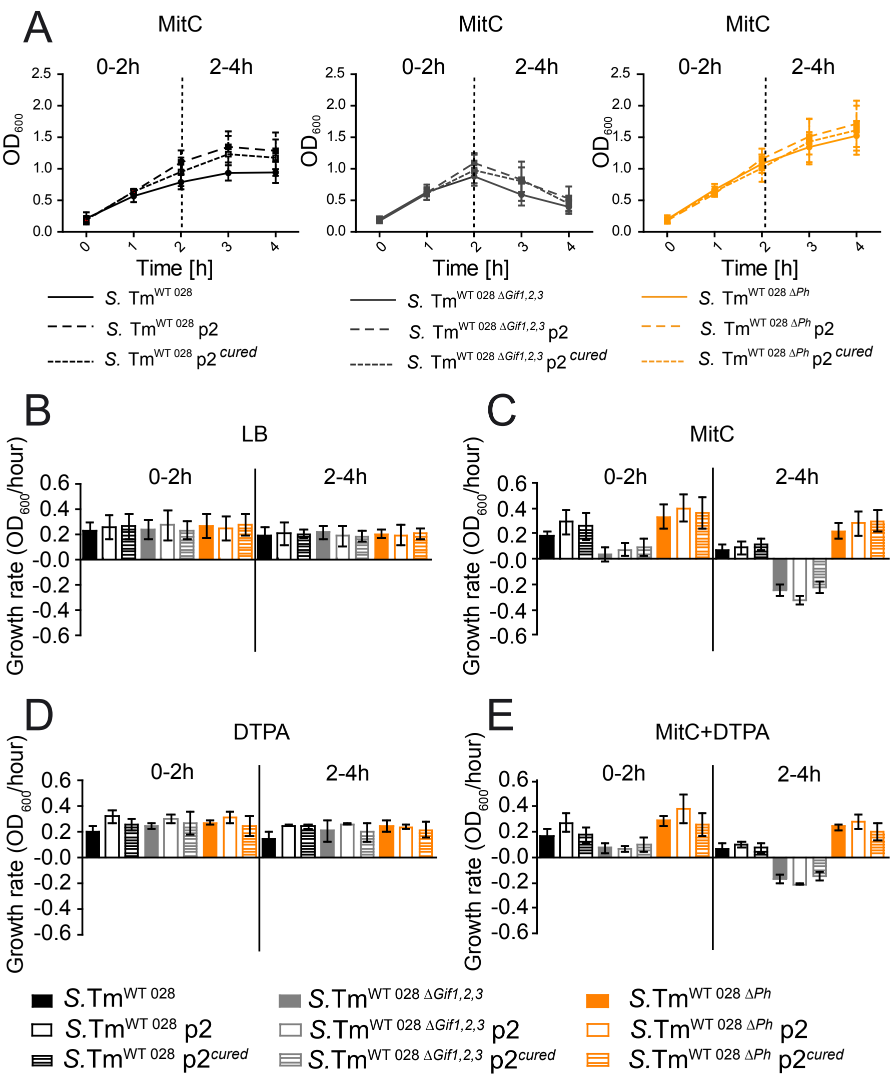

Fig. S3. Net growth rates of S. Tm with and without prophages and pColIb. We compared growth rates of the WT strain (*S.*Tm $^{\text{WT 028}}$), the strain only containing the phage ST64B (*S.*Tm$^{\text{WT 028 Gif1,2,3}}$) and the prophage free strain (*S.*Tm $^{\text{WT 028 Ph}}$). Strains carried either no plasmid, plasmid pColIb (p2) or were again cured of the plasmid (p2^{cured}). Growth rates were determined from changes in OD600 during growth experiments in 96-well plates. (A) As example, growth of all strains over four hours in LB (0.5 μg/ml MitC) is shown. (B-E) From growth curves, the change in biomass per hour was calculated as the slope of the linear regression of OD600 values before (hours 0 - 2) and after (hours 2- 4) lysis induction. (B) LB medium, (C) iron limiting conditions (100 μM DTPA), (D) SOS-inducing stress alone (0.5 μg/ml MitC) or in (E) combination with iron limitation (MitC/DTPA) are shown.
