# Supplementary material for: Evolutionary Stabilization of Cooperative Toxin Production through a Bacterium-Plasmid-Phage Interplay
Source: mBio. 2020 Jul 21;11(4):e00912-20. doi: 10.1128/mBio.00912-20 (PMC7374059; doi:10.1128/mBio.00912-20)
Supplement: TABLE S3 [file mBio.00912-20-st003.pdf]

Table S3. Parameter values used

Name	*Value*	*Definition*	*Reference*
D	$1/h$	dilution rate	
s_0	$10^8\ U/\text{ml}$	inflow concentration *Salmonella*	
e_0	$10^8\ U/\text{ml}$	inflow concentration competitor	
r	$1/h$	growth rate *Salmonella*	(14)
r_e	$1.2/h$	growth rate competitor	[1]
K	$2 \cdot 10^9\ U/\text{ml}$	carrying capacity	(15)
β	$3 \cdot 10^{-8}\,\text{ml}/(U\ h)$	infection rate plasmids	(16)
λ	$3.5 \cdot 10^{-10}\,\text{ml}/(U\ h)$	infection rate phages	(15)
ρ	0.01	fraction of newly infected cells that become lysogenic	(17)
ψ	$8.318 \cdot 10^{14}\,\text{ml}/(h\ \text{mol})$	rate at which colicin kills	[2]
δ_{Stress}	$10^{-3}/h$	rate of lysogenic cells to lyse during stress	(15)
$\delta_{noStress}$	$0/h$	rate of lysogenic cells to lyse during no stress	
$\delta(t)$		equals $\delta_{noStress}$ for no stress conditions, and δ_{Stess} in stress conditions.	
γ_Φ	$0.0044/h$	degradation rate of free phages	(15)
γ_C	$0.044\,/h$	degradation rate of colicin	(14)
L	75	burst size (amplification factor of phages)	(18)
L_C	$1.661 \cdot 10^{-20}\ \text{mol}/U$	amount of colicin released by lysis of cells with plasmids	[3]
ζ_{act}	$2/h$	activation of colicin production (stress)	[4]
ζ_{deAct}	$2/h$	de-activation of colicin production (no stress)	[4]
T	50 h	period of inflow/stress	
$t \in [0, T/2]$		acute infectious conditions (inflow)	

[1] The competitor is assumed to have a higher growth rate than *Salmonella* (in accordance with experimental data).

[2] The parameter ψ is a constant of proportionality. Given a colicin concentration C, the death rate for bacteria is ψC. It was assumed that half of the bacteria die after 5 minutes if considerable amounts of colicin are present, which corresponds to a death rate of $8.318/h$. The colicin concentration in our simulations varies over magnitudes, between zero and maxima around 10^{-12} mol/ml). Often, it is in the range of $10^{-14} - 10^{-15}$mol/ml. Therefore, we choose $\psi = 8.318/(10^{-14}\text{h mol/ml})$.

[3] Based on the assumption that 10,000 molecules of colicin are released by lysis of cells with plasmids.

[4] The reaction time of cells to react to the presence respectively absence of stress is assumed to be about 1/2 h.
