# Supplementary material for: Evolutionary Stabilization of Cooperative Toxin Production through a Bacterium-Plasmid-Phage Interplay
Source: mBio. 2020 Jul 21;11(4):e00912-20. doi: 10.1128/mBio.00912-20 (PMC7374059; doi:10.1128/mBio.00912-20)
Supplement: TEXT S1 [file mBio.00912-20-s0001.pdf]

Supplemental Information

Evolutionary stabilization of cooperative toxin production through a bacterium-plasmid-phage interplay

Stefanie Spriewald[1,2], Eva Stadler[3], Burkhard A. Hense[4], Philipp C. Münch[1,5], Alice C. McHardy[5], Anna S. Weiss[1], Nancy Obeng[1,6], Johannes Müller[3‡], and Bärbel Stecher[1,2‡]

‡ These authors contributed equally

[1] Max-von-Pettenkofer Institute, LMU Munich, Pettenkoferstr. 9a, 80336 Munich, Germany

[2] German Center for Infection Research (DZIF), partner site LMU Munich, Munich Germany

[3] Zentrum Mathematik, Technische Universität München, Munich, Germany

[4] Institute of Computational Biology, Helmholtz Zentrum München Neuherberg, Germany

[5] Department for Computational Biology of Infection Research, Helmholtz Center for Infection Research, Brunswick, Germany

[6] current address: Evolutionary Ecology and Genetics, Zoological Institute, CAU Kiel, Kiel, Germany

Supplemental Text

1. Ecological model of the tripartite interaction of *S.* Tm, the colicin plasmid and a lysogenic phage in the group B colicin system

In order to address the effect of evolutionary forces on the group B colicin system in *S.* Tm, we devised a model based on ordinary differential equations (ODEs), as described in the main text **Figure 3**. The model takes into account *S.* Tm, the plasmid harboring the colicin Ib (*cib*) and colicin Ib immunity (*imm*) genes, a prototype for a functional lysogenic *S.* Tm phage and a competing species (e.g. *commensal E. coli*; **Figure 3**; **Table S3**).

The parameter values used for the model are based on a combination of experimental data, literature and estimations (Obeng, Spriewald and Stecher, unpublished; **Table S3**).

This ecological model was used as the basis to analyze the evolutionary stability of bacterial lysis using adaptive dynamics (see below), from the phages' perspective (with and without colicin production).

The compartments of the ecological model are:

e	competitor (*E. coli*)
s_-^-	*S.* Tm without plasmids and without phages
s_-^+	*S.* Tm without plasmids and with phages
s_+^-	*S.* Tm with plasmids and without phages, not producing colicin but immune to colicin
s_+^+	*S.* Tm with plasmids and with phages, not producing colicin but immune to colicin
s_{+c}^-	*S.* Tm with plasmids and without phages, producing colicin and immune to colicin. No colicin release
s_{+c}^+	*S.* Tm with plasmids and with phages, producing colicin and immune to colicin. Colicin release
Φ	free phages
C	free colicin

The S. Tm population is denoted with s, the superscript indicates the presence (+) or absence (-) of the prophage and the subscript indicates the presence (+) or absence (-) of the plasmid. The c in the subscript indicates that the bacterium is producing colicin.

The dynamics of the system are given by the following ODEs:

$$\begin{aligned} e' &= D(e_0(t) - e) + r_e e(1 - p/K) - \psi\, C\, e \\ s_-^{-\prime} &= D(s_0(t) - s_-^-) + r\, s_-^-(1 - p/K) - \beta(s_+^- + s_+^+ + s_{+c}^- + s_{+c}^+)s_-^- \end{aligned}$$

$$
\begin{aligned}
& -\lambda\,\Phi\,s_{-}^{-} - \psi\,C\,s_{-}^{-} \\
s_{+}^{-\,\prime} &= -D\,s_{+}^{-} \qquad + r\,s_{+}^{-}(1-p/K) + \beta(s_{+}^{-}+s_{+}^{+}+s_{+c}^{-}+s_{+c}^{+})s_{-}^{-} \\
& -\lambda\,\Phi\;s_{+}^{-} - S(t)\,\zeta_{act}s_{+}^{-} + (1-S(t))\,\zeta_{deAct}s_{+c}^{-} \\
s_{+c}^{-\,\prime} &= -D\,s_{+c}^{-} \qquad + r\,s_{+c}^{-}(1-p/K) \\
& -\lambda\,\Phi\;s_{+}^{-} + S(t)\,\zeta_{act}s_{+}^{-} - (1-S(t))\,\zeta_{deAct}s_{+c}^{-} \\
s_{-}^{+\,\prime} &= -D\,s_{-}^{+} \qquad + r\,s_{-}^{+}(1-p/K) - \beta(s_{+}^{-}+s_{+}^{+}+s_{+c}^{-}+s_{+c}^{+})s_{-}^{+} \\
& +\rho\,\lambda\,\Phi\,s_{-}^{-} - \psi\,C\,s_{-}^{+} - \delta(t)s_{-}^{+} \\
s_{+}^{+\,\prime} &= -D\,s_{+}^{+} \qquad + r\,s_{+}^{+}(1-p/K) + \beta(s_{+}^{-}+s_{+}^{+}+s_{+c}^{-}+s_{+c}^{+})s_{-}^{+} \\
& +\rho\,\lambda\,\Phi\,s_{+}^{-} - \delta(t)s_{+}^{+} - S(t)\,\zeta_{act}s_{+}^{+} + (1-S(t))\,\zeta_{deAct}s_{+c}^{+} \\
s_{+c}^{+\,\prime} &= -D\,s_{+c}^{+} \qquad + r\,s_{+c}^{+}(1-p/K) \\
& +\rho\,\lambda\,\Phi\,s_{+c}^{-} - \delta(t)s_{+c}^{+} + S(t)\,\zeta_{act}s_{+}^{+} - (1-S(t))\,\zeta_{deAct}s_{+c}^{+} \\
\Phi' &= -D\,\Phi + L\,\delta(t)(s_{-}^{+}+s_{+}^{+}+s_{+c}^{+}) + (1-\rho)\,L\,\lambda\,\Phi\,(s_{-}^{-}+s_{+}^{-}+s_{+c}^{-}) - \gamma_{\Phi}\,\Phi \\
C' &= -D\,C + L_C(\delta(t)s_{+c}^{+} + (1-\rho)\,\lambda\,\Phi\,s_{+c}^{-}) - \gamma_C\,C,
\end{aligned}
$$

where

$$
p = s_{-}^{-} + s_{+}^{-} + s_{+c}^{-} + s_{-}^{+} + s_{+}^{+} + s_{+c}^{+} + e,
$$

$S(t) \in \{0,1\}$ indicates stress ($s=1$) or no-stress ($s=0$) conditions, and $\delta(t) = \delta_{noStress}$ if there is no stress, and $\delta(t) = \delta_{Stress}$ for stress conditions. During acute infection conditions there is inflow at rate s_0 (into s_{-}^{-}) resp. e_0 (into e), with s_0, e_0 stated in **Table S3**. Stress signal and acute infection conditions are periodic with period T. Acute infection is always present for $t \in [0, T/2]$. For a reliable stress signal, $S(t) = 1$ in $t \in [0, T/2]$ and $S(t) = 0$ for the remaining part of the period. For non-reliable stress signals, $S(t) = 1$ in $t \in [0, T/2] \cup [15\,T/24, 17\,T/24]$

and zero in the complementary part of the period.

2. Adaptive dynamics of the evolutionary stability of lysis in the group B colicin system

We investigated the evolutionary stability of lysis from the phages' perspective i.e. investigated the fate of mutant phages with higher or lower lysis rate via adaptive dynamics. For this procedure, mutant phages (with altered δ_{Stress} or ρ) are introduced into the ecological model. These mutants may appear as free infectious phages, but also as prophages in *S.* Tm.

Thus, we add the following compartments to the ecological model:

s_-^m	*S.* Tm without plasmids and with mutant phages
s_+^m	*S.* Tm with plasmids and with mutant phages, not producing but immune to colicin
s_{+c}^m	*S.* Tm with plasmids and with mutant phages, producing and immune to colicin. Colicin release
Φ_m	free mutant phages

The presence of a mutant phage is now symbolized by a superscript m instead of the superscript $+$ that is used for the wild type phages. Free mutant phages are denoted by Φ_m.

We augment the equations accordingly. Here, $\delta_m(t)$ and ρ_m denote the respective parameters of the mutants:

$$\begin{aligned}
e' &= D(e_0(t) - e) + r_e e(1 - p/K) - \psi\, C\, e \\
{s_-^-}' &= D(s_0(t) - s_-^-) + r\, s_-^-(1 - p/K) - \beta(s_+^- + s_+^+ + s_+^m + s_{+c}^- + s_{+c}^+ + s_{+c}^m)\, s_-^- \\
&\quad -\lambda\, \Phi\, s_-^- - \lambda\, \Phi_m\, s_-^- - \psi\, C\, s_-^- \\
{s_+^-}' &= -D\, s_+^- + r\, s_+^-(1 - p/K) + \beta(s_+^- + s_+^+ + s_+^m + s_{+c}^- + s_{+c}^+ + s_{+c}^m) s_-^- \\
&\quad -\lambda\, \Phi\; s_+^- - \lambda\, \Phi_m\; s_+^- - S(t)\, \zeta_{act} s_+^- + (1 - S(t))\, \zeta_{deAct} s_{+c}^-
\end{aligned}$$

$$
\begin{aligned}
{s^{-}_{+c}}' &= -D\, s^{-}_{+c} + r\, s^{-}_{+c}(1-p/K) \\
&\quad -\lambda\,\Phi\ s^{-}_{+} - \lambda\,\Phi_m\ s^{-}_{+} + S(t)\,\zeta_{act} s^{-}_{+} - (1-S(t))\,\zeta_{deAct} s^{-}_{+c} \\
{s^{+}_{-}}' &= -D\, s^{+}_{-} + r\, s^{+}_{-}(1-p/K) - \beta(s^{-}_{+} + s^{+}_{+} + s^{m}_{+} + s^{-}_{+c} + s^{+}_{+c} + s^{m}_{+c}) s^{+}_{-} \\
&\quad +\rho\,\lambda\,\Phi\, s^{-}_{-} - \psi\, C\, s^{+}_{-} - \delta(t) s^{+}_{-} \\
{s^{+}_{+}}' &= -D\, s^{+}_{+} + r\, s^{+}_{+}(1-p/K) + \beta(s^{-}_{+} + s^{+}_{+} + s^{m}_{+} + s^{-}_{+c} + s^{+}_{+c} + s^{m}_{+c}) s^{+}_{-} \\
&\quad +\rho\,\lambda\,\Phi\, s^{-}_{+} - \delta(t) s^{+}_{+} - S(t)\,\zeta_{act} s^{+}_{+} + (1-S(t))\,\zeta_{deAct} s^{+}_{+c} \\
{s^{+}_{+c}}' &= -D\, s^{+}_{+c} + r\, s^{+}_{+c}(1-p/K) \\
&\quad +\rho\,\lambda\,\Phi\, s^{-}_{+c} - \delta(t) s^{+}_{+c} + S(t)\,\zeta_{act} s^{+}_{+} - (1-S(t))\,\zeta_{deAct} s^{+}_{+c} \\
\Phi' &= -D\,\Phi + L\,\delta(t)(s^{+}_{-} + s^{+}_{+} + s^{+}_{+c}) + (1-\rho)\, L\,\lambda\,\Phi\,(s^{-}_{-} + s^{-}_{+} + s^{-}_{+c}) \\
&\quad -\gamma_{\Phi}\,\Phi \\
C' &= -D\, C + \ L_C(\delta(t) s^{+}_{+c} + (1-\rho)\,\lambda\,\Phi\, s^{-}_{+c} + \delta_m(t) s^{m}_{+c} \\
&\quad +(1-\rho_m)\,\lambda\,\Phi\, s^{-}_{+c}) - \gamma_C\, C \\
{s^{m}_{-}}' &= -D\, s^{m}_{-} + r\, s^{m}_{-}(1-p/K) - \beta(s^{-}_{+} + s^{+}_{+} + s^{m}_{+} + s^{-}_{+c} + s^{+}_{+c} \\
&\quad +s^{m}_{+c})\, s^{m}_{-} + \rho_m\,\lambda\,\Phi_m\, s^{-}_{-} - \psi\, C\, s^{m}_{-} - \delta_m(t) s^{m}_{-} \\
{s^{m}_{+}}' &= -D\, s^{m}_{+} + r\, s^{m}_{+}(1-p/K) + \beta(s^{-}_{+} + s^{+}_{+} + s^{m}_{+} + s^{-}_{+c} + s^{+}_{+c} \\
&\quad +s^{m}_{+c})\, s^{m}_{-} + \rho_m\,\lambda\,\Phi_m\, s^{-}_{+} - \delta_m(t) s^{m}_{+} - S(t)\,\zeta_{act} s^{m}_{+} \\
&\quad +(1-S(t))\,\zeta_{deAct} s^{m}_{+c} \\
{s^{m}_{+c}}' &= -D\, s^{m}_{+c} + r\, s^{m}_{+c}(1-p/K) \\
&\quad +\rho_m\,\lambda\,\Phi_m\, s^{-}_{+c} - \delta_m(t) s^{m}_{+c} + S(t)\,\zeta_{act} s^{m}_{+} - (1-S(t))\,\zeta_{deAct} s^{m}_{+c} \\
\Phi_m' &= -D\,\Phi_m + L\,\delta_m(t)(s^{m}_{-} + s^{m}_{+} + s^{m}_{+c}) \\
&\quad +(1-\rho_m)\, L\,\lambda\,\Phi_m\,(s^{-}_{-} + s^{-}_{+} + s^{-}_{+c}) - \gamma_{\Phi}\,\Phi_m,
\end{aligned}
$$

where

$$p = s_-^- + s_+^- + s_{+c}^- + s_-^+ + s_-^m + s_+^+ + s_+^m + s_{+c}^+ + s_{+c}^m + e,$$

and $\delta_m(t) = \delta_{noStress} = 0$ if there is no stress, and $\delta_m(t) = \delta_{Stress,m}$ else.

In absence of the mutant the solution for the resident is periodic. We introduce mutants at very small numbers and measure the Lyapunov exponent. If the Lyapunov exponent is positive, the mutant can spread, if it is negative, the mutant dies out. It has been checked numerically for a sample of parameter values that a rare mutant that can spread eventually outcompetes the resident, such that the resident dies out and the mutant becomes the new resident. This numerical analysis indicates that the present model satisfies the prerequisites for adaptive dynamics. The pairwise invasibility plots show the sign of the Lyapunov coefficient for different parameter values. The ESS can be read off the pairwise invasibility plots, as discussed in the main text (**Figure 4**).

Numerical procedure to determine the Lyapunov exponent: The ODE has been solved for a burn-in phase of four periods where all mutant compartments are taken to zero. Then, rare mutants are added; the initial density is chosen proportional to the corresponding wild life densities, scaled in such a way that the total inital mutant population was $10^{-8}U$. A further burn-in phase of 8.6 (resp. 2.6 periods for the model without colicin) periods was used. Subsequently the Lyapunov coefficient has been measured during two periods. If the Lyapunov coefficient has been smaller than zero, we decided that the mutant is not able to invade, if the coefficient was larger than zero, we decided that the mutant can invade.

For the analysis of the model without colicin production, we took $L_C = 0$.

We have only investigated evolutionary stability of lysis from the phages' perspective, i.e. investigated the fate of mutant phages with higher or lower lysis rate. It is imaginable that also

bacterial mutants arise with mutations affecting the lysis rate by the phage (e.g. in signaling pathways of the SOS response). Mutations in this pathway would on the one hand influence the kinetics of prophage-mediated cell lysis. On the other hand, other genes regulated by the SOS-response, such as DNA-repair mechanisms and cell division would be affected. Therefore, more parameters would be required to model this complex trade-off between benefits and costs of bacterial mutations affecting the outcome of prophage-induction.

Supplemental Movies

Movie S1: CellAsic® live cell microscopy of *S.* Tm$^{\mathbf{WT}}$p$^{\mathbf{P}_{cib\ gfp}}$. Using the CellASIC® ONIX microfluidic system (Millipore), microcolonies of *S.* Tm^{WT}p$^{\mathrm{P}_{cib\ gfp}}$ were initially grown in LB for 3 h. Subsequently, the media was exchanged against LB supplemented with DTPA (100 μM) to induce *cib* (*gfp*) expression only. 20 min later, LB media containing DTPA (100 μM) and MitC (0.5 μg/ml) was applied, to induce full expression of *cib* (*gfp*) and in addition prophage-mediated cell lysis for 20 min. Afterwards, the media was changed back to LB supplemented with DTPA (100 μM) only for the remaining time. *(A)* Merged brightfield- and GFP-channel. *(B)* GFP channel only.

Movie S2: CellAsic® live cell microscopy of *S.* Tm$^{\mathbf{Ph}}$p$^{\mathbf{P}_{cib\ gfp}}$. Using the CellASIC® ONIX microfluidic system (Millipore), microcolonies of *S.* Tm^{Ph}p$^{\mathrm{P}_{cib\ gfp}}$ were initially grown in LB for 3 h. Subsequently, the media was exchanged against LB supplemented with DTPA (100 μM) to induce *cib* (*gfp*) expression only. 20 min later, LB media containing DTPA (100 μM) and MitC (0.5 μg/ml) was applied for 20 min, to induce full expression of *cib* (*gfp*) and to show the failure of cell lysis in the absence of prophages. Afterwards, the media was changed back to LB supplemented with DTPA (100 μM) only for the remaining time. *(A)* Merged brightfield- and GFP-channel. *(B)* GFP channel only.

Movie S3: CellAsic® live cell microscopy of *S.* Tm$^{lysST::T7\ pol}$p$^{\mathbf{P}_{T7\ rfp}\mathbf{P}_{cib\ gfp}}$. Using the CellASIC® ONIX microfluidic system (Millipore), microcolonies of *S.* Tm$^{lysST::T7\ pol}$p$^{\mathrm{P}_{T7\ rfp}\mathrm{P}_{cib\ gfp}}$ were initially grown in LB for 3 h. Subsequently, the media was exchanged against LB supplemented with DTPA (100 μM) to induce *cib* (*gfp*) expression only.

20 min later, LB media containing DTPA (100 μM) and MitC (0.5 μg/ml) was applied for 20 min, to induce full expression of *cib* (*gfp*) and ST64B prophage lysis gene induction (*rfp*). Afterwards, the media was changed back to LB supplemented with DTPA (100 μM) only for the remaining time. *(A)* Merged GFP- and RFP-channel. *(B)* GFP (cib expression) channel only *(C)* RFP (ST64B prophage lysis gene expression) channel only.

References

[1] Hoiseth SK, Stocker BAD. Aromatic-dependent Salmonella Typhimurium are non-virulent and effective as live vaccines. Nat. 1981; 291(5812): 238–239.

[2] Figueroa-Bossi N, Uzzau S, Maloriol D, Bossi L. Variable assortment of prophages provides a transferable repertoire of pathogenic determinants in Salmonella. Mol. 2001; 39(2):260–271.

[3] Spriewald S, Glaser J, Beutler M, Koeppel MB, Stecher B. Reporters for Single-Cell Analysis of Colicin Ib Expression in Salmonella enterica Serovar Typhimurium. PloS one 2015; 10(12):e0144647.

[4] Alonso A, Pucciarelli MG, Figueroa-Bossi N, Garcia-del Protillo F. Increased excision of the Salmonella prophage ST64B caused by a deficiency in Dam methylase. Journal of bacteriology 2005; 187(23):7901–7911.

[5] Nedialkova LP, Sidstedt M, Koeppel MB, Spriewald S, Ring D, Gerlach RG, Bossi L, Stecher B. Temperate phages promote colicin-dependent fitness of Salmonella enterica serovar Typhimurium. Environ. 2016; 18(5):1591–1603.

[6] Figueroa-Bossi N Bossi L. Resuscitation of a defective prophage in Salmonella co-cultures. Journal of bacteriology 186(12):4038-4041.

[7] Moller AK, et al. An Escherichia coli MG1655 lipopolysaccharide deep-rough core mutant grows and survives in mouse cecal mucus but fails to colonize the mouse large intestine. Infection and immunity 71(4):2142-2152.

[8] Wang RF, Kushner SR. Construction of versatile low-copy-number vectors for cloning, sequencing and gene expression in Escherichia coli. Gene 1991; 100:195–199.

[9] Datsenko KA, Wanner BL. One-step inactivation of chromosomal genes in Escherichia coli K-12 using PCR products. Proceedings of the National Academy of Sciences of the United States of America 2000; 97(12):6640–6645.

[10] Bender JK, Wille T, Blank K, Lange A, Gerlach RG. LPS structure and PhoQ activity are important for Salmonella Typhimurium virulence in the Gallleria mellonella infection model. PloS one 2013; 8(8):e73287.

[11] Cherepanov PP, Wackernagel W. Gene disruption in Escherichia coli: TcR and KmR cassettes with the option of Flp-catalyzed excision of the antibiotic-resistance determinant. Gene 1995; 158(1):9–14.

[12] Stecher B, Hapfelmeier S, Müller C, Kremer M, Stallmach T, Hardt WD. Flagella and chemotaxis are required for efficient induction of Salmonella enterica serovar Typhimurium colitis in streptomycin-pretreated mice. Infect. 2004; 72(7):4138–4150.

[13] Nedialkova LP, Denzler R, Koeppel MB, Diehl M, Ring D, Wille T, Gerlach RG, Stecher B. Inflammation fuels colicin Ib-dependent competition of Salmonella serovar Typhimurium and E.coli in enterobacterial blooms. PloS pathogens 2014; 10(1).

[14] van Raay K, Kerr B. Toxins go viral: Phage-encoded lysis releases group B colicins. Environ. 2016; 18(5):1308–1311.

[15] Brown SP, Le Chat L, de Paepe M, Taddei F. Ecology of microbial invasions: Amplification allows virus carriers to invade more rapidly when rare. Curr. 2006; 16(20):2048–2052.

[16] Wan Z, Varshavsky J, Teegala S, McLawrence J, Goddard NL. Measuring the rate of conjugal plasmid transfer in a bacterial population using quantitative PCR. Biophys. 2011; 101(1):237–244.

[17] Court DL, Oppenheim AB, Adhya SL. A new look at bacteriophage lambda genetic networks. Journal of Bacteriology 2007; 189(2):298–304.

[18] Alexander M, Bloom BR, Hopwood DA, Hull R, Iglewski BH, Laskin AI, Oliver SG, 26 332 Schaechter M, Summers WC, Lederberg J. Encyclopedia of Microbiology: Four-Volume 333 Set. 2nd ed. Burlington: Elsevier; 2000. http://gbv.eblib.com/patron/FullRecord. 334 aspx?p=305562.
