# Supplementary material for: Evolutionary Stabilization of Cooperative Toxin Production through a Bacterium-Plasmid-Phage Interplay
Source: mBio. 2020 Jul 21;11(4):e00912-20. doi: 10.1128/mBio.00912-20 (PMC7374059; doi:10.1128/mBio.00912-20)
Supplement: TABLE S4 [file mBio.00912-20-st004.pdf]

Table S4. Gene families for group A and B colicins* and phage lysis genes.

Grouping	*Name*	*ID*
Group A colicin	Cluster: Colicin-K	UniRef50_Q47502
	Cluster: Colicin-N	UniRef50_P08083
	Cluster: Colicin-E1	UniRef50_P02978
	Cluster: Colicin-E9	UniRef50_P09883
Group B colicin	Cluster: Colicin-D	UniRef50_P17998
	Cluster: Colicin-Ia/Ib	UniRef50_P06716
	Cluster: Colicin-M	UniRef50_P05820
	Cluster: Colicin-B	UniRef50_P05819
lysis genes	endolysin + lysome; Phage_lysozyme	PF00959
	canonical holin; Bacteriophage P21 holin S	PF04971
	canonical endolysin; Family: Muraidase; N-acetylmuramidase	PF11860
	i-spanin; Family: Phage_lysis; Bacteriophage Rz lysis protein	PF03245
	o-spanin; Family: Rz1; Lipoprotein Rz1 precursor	PF06085

* We further screened for Cluster "Colicin-E5" (UniRef50_UPI00022A9A6A) and Cluster "Colicin FY" (UniRef50_H8YUQ0) but did not find any match exceeding the thresholds.
